# Modelling COVID-19 transmission dynamics in Laos under non-pharmaceutical interventions, vaccination, and replacement of SARS-CoV-2 variants

**DOI:** 10.1186/s44263-024-00069-y

**Published:** 2024-06-17

**Authors:** Xu-Sheng Zhang, Hong Luo, Andre Charlett, Daniela DeAngelis, Wei Liu, Peter Vickerman, Mark Woolhouse, Linxiong Wu

**Affiliations:** 1 Statistics, Modelling and Economics, Data, Analytics & Surveillance, UK Health Security Agency, London, UK; 2 Education College, Yunnan University, Kunming, Yunnan People’s Republic of China; 3 Medical Research Council Biostatistics Unit, University Forvie Site, Robinson Way, Cambridge, UK; 4 School of Public Health, Kunming Medical University, Kunming, Yunnan People’s Republic of China; 5 Population Health Sciences, University of Bristol, Bristol, UK; 6 Usher Institute, University of Edinburgh, Edinburgh, UK; 7 Yunnan Provincial Key Laboratory of Public Health and Biosafety, Kunming, Yunnan People’s Republic of China

**Keywords:** Bayesian estimation, Control measures, COVID-19, SARS-CoV-2, Oxford Government COVID-19 policy index, Google mobility, Non-pharmaceutical interventions (NPIs), Modelling, Variant, Vaccination

## Abstract

**Background:**

Understanding how the COVID-19 pandemic evolved under control measures is crucial to tackle the SARS-CoV-2 virus spread. Laos, a country bordering China but with late occurrence and low burden of COVID-19 compared to its neighbouring countries, was used for a case study.

**Methods:**

A transmission model with disease reporting was proposed to investigate the impact of control measures on the SARS-CoV-2 virus spread in Laos from April 2021 to May 2022. It was assumed that the transmission rate changed with people’s behaviours, control measures and emerging variants; susceptibility decreased with vaccination and infection. Bayesian inference was used for model calibration to data of confirmed cases, deaths, and recoveries, and the deviance information criterion was used to select the best model variant.

**Results:**

Our model including Non-pharmaceutical interventions (NPIs), behaviour change, vaccination, and changing variants well explained the three waves in Laos. The Alpha variant was estimated to have a basic reproduction number of 1.55 (95% CrI: 1.47–1.64) and was replaced by the Delta variant from September 2021 which was 1.88 (95% CrI: 1.77–2.01) times more transmissible; the Delta variant was replaced by Omicron variant from March 2022 which was 3.33 (95% CrI: 2.84–3.74) times more transmissible. The Delta variant was the most severe with a case fatality rate of 1.05% (95% CrI: 0.96–1.15%) while the Alpha variant and Omicron variant were much milder. The ascertainment rate was low and variable: first decreasing from 13.2 to 1.8% by 23 May 2021, and then increasing to 23.4% by 15 March 2022. Counterfactual simulations indicated that vaccination played strong roles in reducing infections even under the emergence of immune escape variants while behaviour change delayed but might not flatten the peak of outbreaks.

**Conclusions:**

The three waves of Laos’ epidemics were due to the invasion of more transmissible and immune escape variants that affected the herd immunity built via vaccination and infection. Even with immunity waning and the escape of new variants, vaccination was still the major contributor to control COVID-19 and combining behaviour changes and vaccination would best suppress future outbreaks of COVID-19.

**Supplementary Information:**

The online version contains supplementary material available at 10.1186/s44263-024-00069-y.

## Background

Since its emergence in Wuhan City, China, in December 2019, COVID-19 caused by the SARS-CoV-2 virus quickly spread to the world. As of 12 May 2022, over 514 million confirmed cases and over 6 million deaths have been reported globally (https://www.who.int/publications/m/item/weekly-epidemiological-update-on-covid-19---11-may-2022). SARS-CoV-2 is a newly emerging virus, and at its early stage, there was no vaccine or drug to protect people. Non-pharmaceutical interventions (NPIs), such as internal containment and closure, travel restrictions, economic measures, face mask ordinances, and quarantine, were widely used to fight COVID-19 [1, 2]. NPIs played an important role in controlling SARS-CoV-2 transmission by reducing the contact rates between people. Vaccine was started to roll out at the end of 2020 [3] and has played a crucial role in reducing symptomatic infections and severe cases [3, 4]. As new variants of concern (VOC) were quickly evolved and circulated, the world had been experiencing several waves even under the large scale of vaccination because the new variants became more transmissible and evaded immunity acquired via vaccines and infections [5].

VOC were new genetic versions of the virus with increased transmissibility, changed virulence, or decreased effectiveness of mitigation measures, vaccines, or treatments. The most widely circulating variants were Alpha, Delta, and Omicron [6, 7]. Alpha (B.1.1.7) was the first of the highly publicised variants and was first identified in November 2020 in the UK [8, 9]. Delta (B.1.617.2) was first identified in spring 2021 in India and rapidly replaced other variants, achieving global dominance by summer 2021 [10]. The highly divergent Omicron variant (B.1.1.529) was identified in mid-November 2021 and quickly dominated in Europe and North America by late December 2021 [11]. Although these variants mostly replaced one by another and there were periods of co-circulation, co-infection appeared not common [12]. The estimation showed the relationships: Omicron > Delta > Alpha in transmissibility [13] and Omicron < Delta < Alpha in vaccine effectiveness (VE) [5, 7, 14–16], but in their severity: Delta > Omicron, Delta > Alpha [6, 7, 17].

**Fig. 1:**
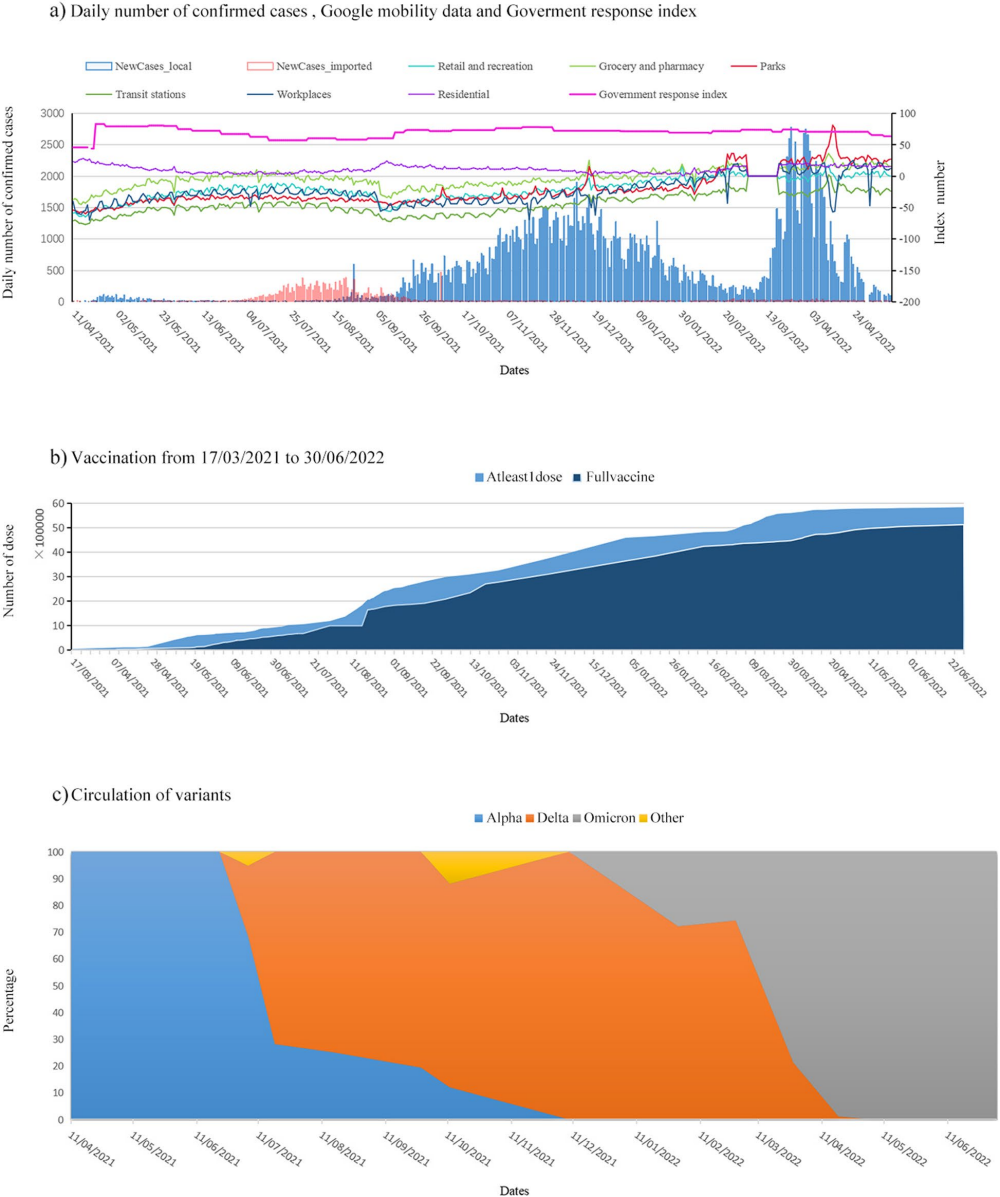
COVID-19 outbreaks in Laos: **a** the temporal changes in local and imported cases along with the control measures implemented in Laos and Google mobility data during the period from 11 April 2021 to 12 May 2022, **b** vaccines delivered, and **c** circulation of the variants of the SARS-CoV-2 virus

Lao Peoples Democratic Republic (shortened as Laos in the following) has a population of 7,389,060. Laos has borders with Vietnam to the east, Cambodia to the south, Thailand to the west, and Myanmar and China to the north. Laos has limited public health infrastructure but reported a particularly low burden of disease at the early stage of the pandemic [18] (Additional file 1: Table S1). Despite bordering China, it was the last country in Southeast Asia to report confirmed cases (Additional file 1: Fig. S1) [18, 19]: 23 cases were identified by September 2020 with minimal SARS-CoV-2 circulation [20]. After 1 year with no reported cases, COVID-19 came back on 11 April 2021 [18], and increased quickly due to the celebration of the Laos New Year on 14–16 April 2021 but was suppressed within 1 month (Fig. 1a) due to quick actions from 22 April 2021 and rapid national lockdown and closure of all land borders resulted from the first COVID-19–related death reported on 9th May 2021 (see Additional file 1: Tables S1 and S2). As control measures relaxed, cases increased from August 2021 and reached a peak before January 2022. After staying at a low level of incidence in February 2022, COVID-19 resurged from March 2022 but decreased from the start of April 2022. To control the spread of COVID-19, vaccines were deployed in Laos from March 2021 (Fig. 1b). Along with the change in the size of reported cases, the VOC of SARS-CoV-2 that were circulated also altered (Fig. 1c).

These changes in incidence should be driven by control measures such as NPIs, vaccines, circulating variants and voluntary behaviour change. Quantifying their impacts is important to controlling COVID-19. The available information about people’s behaviours, control measures, and outbreak data, provided by, for example, Google mobility data [21], Oxford Policy tracker [22, 23], and Our World in data [24, 25], enables the research on the determinants of COVID-19 waves. Mathematical models have long been used to investigate the spread of infectious diseases [26] and have become an important component of global responses to the COVID-19 pandemic [1, 27–29]. Although COVID-19 is now under control, it is vital and of immense value to learn about its transmission and epidemic control under different populations and cultures from what happened during the pandemic. In this study, we incorporated NPIs and voluntary behaviour changes based on Google mobility data and Government Response indexes, incidence data (Fig. 1a), vaccination data (Fig. 1b) and sequencing data (Fig. 1c) into a transmission model to investigate how the response policy, vaccination, and people’s protective behaviours shaped the COVID-19 epidemics in Laos under the replacement of SARS-CoV-2 variants. Our analyses showed that the COVID-19 epidemics in Laos were collectively driven by behavioural change, vaccination, and alternation of variants; and vaccination was the major contributor suppressing COVID-19 spread even under immunity waning and escape of new variants.

## Methods

### Data

Data of confirmed cases, deaths, and recoveries (Fig. 1a and Additional file 1: Fig. S1) are extracted from the Ministry of Health of Laos [30]. As of 12 May 2022, 209,028 confirmed cases and 752 deaths due to COVID-19 were reported in Laos. Up to 10 April 2021, only 49 sporadic cases were reported and from then it increased rapidly (Additional file 1: Fig. S1). In view of these, the study period was chosen from 11 April 2021 to 12 May 2022. To smooth the irregular reporting patterns, a 7-day rolling averages were used of the originally reported data of confirmed cases and deaths.

Google’s mobility reports [21] were used to assess the temporal contact rate among people in Laos (Additional file 1: Fig. S1; Equation (S10a)). It showed how visits to six locations changed in each geographic region, with a baseline day defined by the median value over the 5-week period from 3 January to 6 February 2020. Government response index, provided by Oxford COVID-19 Government Response Tracker (OxCGRT) [22, 23], was also used to reflect the effect of NPIs on transmission dynamics (Additional file 1: Fig. S1; Equation (S10b)); it is a composite measure based on 16 indicators. Vaccination data were downloaded from Our World in data (Fig. 1b) [24, 25]. Laos rolled out vaccines from March 2021 including six vaccines: Johnson & Johnson, Pfizer/BioNTech, Oxford/AstraZeneca, Sinopharm/Bejing, Sinovac, Sputnik Light and Sputnik V. As of 12 May 2022, 5,791,016 (79.6%) of the total population had been vaccinated with at least one dose of COVID-19 vaccine, with 4,977,532 (68.4%) fully vaccinated (Fig. 1b).

Sequence data of the SARS-CoV-2 virus [31] (Fig. 1c) showed that only the Alpha variant was circulated from April to the end of June 2021. The Delta variant was imported on 7th of July 2021 and became predominant from the middle of August 2021. The Omicron variant emerged from importation on the 1st of February 2022 and has been circulated within the community since early March 2022.

### Model and methods

**Fig. 2:**
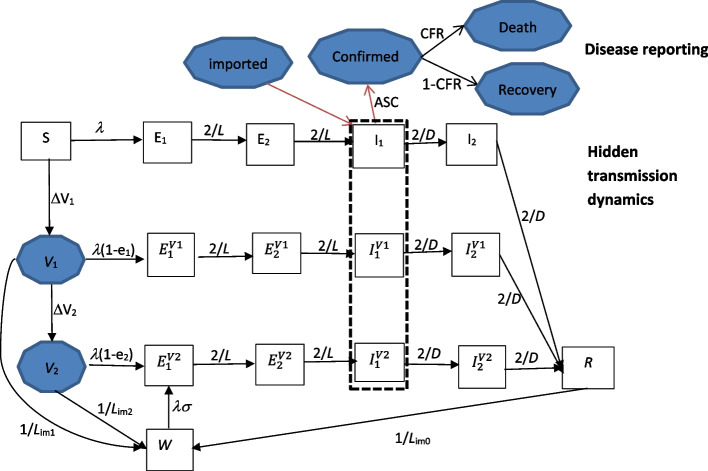
The schematic of the transmission model and disease reporting process. The fifteen rectangle boxes represented the hidden transmission processes, and the six shaded circles represented the quantities upon which we made observations, importation, and the numbers of people having had at least one dose of vaccine (*V*_1_) and fully vaccinated (*V*_2_). Within the transmission model, individuals started as *susceptible* to infection; under the force of infection *λ*, they were exposed to SARS-CoV-2. When *exposed* they became latently infected but not yet infectious; after a latent period of *L*, they then became *infectious*, and after an infectious period of *D*, they *recovered* and became immune; after an immunity period of *L*_im0_, they transferred to a *waned* compartment *W* on which they became partial susceptible to infection. The latent period and infectious period were divided into two equal parts (i.e. *E*_1_(*t*) and *E*_2_(*t*), and *I*_1_(*t*) and *I*_2_(*t*)) respectively to allow their distributions to follow gamma distribution. Susceptible individuals were vaccinated at rate ΔV_1_ and further fully vaccinated at rate ΔV_2_; the vaccinated can be either infected as the susceptible but at reduced force of infection; or waned to compartment *W*. Within the disease reporting process, the infectious individuals were *confirmed* at a proportion (ASC). A proportion (CFR) of the confirmed died and the rest recovered

The model schematic is shown in Fig. 2 with two components: transmission dynamics with vaccination and replacement of SARS-CoV-2 variants, and disease reporting (see Additional file 1: technical details of the “Model and methods” section for details). During the transmission process, a susceptible person contracts the infection from infectious persons and enters the latent class; after a latent period, the exposed person progresses to become infectious, before recovering or dying. The vaccinated acquire partial protection against infection and can be infected at a reduced rate of infection. Immunity acquired via infection or vaccination wanes after an immunity duration. People were assumed to mix randomly by ignoring the effects of population structure and geographical heterogeneity.

The Alpha, Delta, and Omicron variants of the SARS-CoV-2 virus have been circulated and replaced one by another consequently in Laos from 11 April 2021 to 12 May 2022 (Fig. 1c). They differed in transmissibility, severity, and immunity evasion [3, 10, 15, 17]. To reflect temporal changes caused by the alternation of variants, the parameters such as VE, risk of re-infection of the waned, relative infectiousness of the vaccinated, transmissibility, and the case fatality rate were assumed to change in the sigmoidal function way (see Additional file 1: Equations (S3–S9)).

Temporal changes in contact rates recorded by Google mobility data [21] or incurred by the Government response index [22, 23] were incorporated into the effective contact rate (see Additional file 1: Equation (S10)). It should be noted that both data cannot fully reflect the changes in contact rate. For example, behaviour changes such as personal hygiene, face mask wearing, and social distancing were not included in Google mobility data; Government response index cannot tell the extent of compliance to which people followed control policies. Government response index directly defined the NPIs while Google mobility data under the NPIs were expected to reflect the behaviour changes due to the NPIs to some extent, and there was a positive association between them ([32], Fig. 1a). Although both data were assumed to be similarly able to reflect the impact on the transmission of COVID-19, they were used in different models and the better one was chosen for investigating the transmission dynamics in Laos.

The interactions among variants and their interactions with people’s behaviours and immunity were modelled indirectly by the temporal changes in the relevant epidemiological characteristics. Bayesian inference was used to calibrate the model to observational data of confirmed cases, death, and recovered. Deviance information criterion (DIC) [33] was used to compare different model variants. The equations and details of the model were given in Additional file 1: Technical details of Model and Methods. In view of the uncertainty in VE and duration of immunity (see Additional file 1: Tables S4 and S5), sensitivity analyses were conducted under six different scenarios to assess the robustness of model calibration and parameter estimation (see Additional file 1: Sensitivity analyses).

## Results

**Fig. 3:**
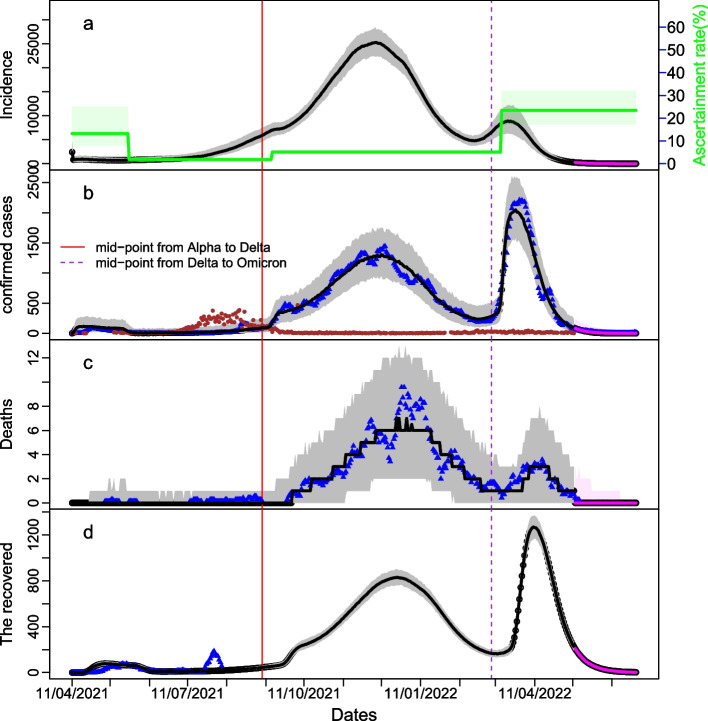
Model estimation of infections and model fit to daily numbers of new local cases, deaths, and recoveries during COVID-19 outbreaks in Laos from 11 April 2021 to 12 May 2022 under the contact model via Google mobility. **a** The daily number of new local infections, **b** the daily number of new local confirmed cases, **c** daily number of new deaths, and **d** daily number of new recovered. The thick black line represented median predictions and grey shading was their 95% credible intervals, with the blue triangles representing the 7-day averages of observational data. The green line in panel (**a)** represented the ascertainment rate which changed at three turning points. The brown dots in panel (**b**) represented the daily numbers of imported cases. The red and purple vertical lines stood for the midpoints of the transition from the Alpha to Delta variant and from the Delta to Omicron variant of the SARS-CoV-2 virus, respectively. The recovery data in panel (**d**) were available up to day 116 (i.e. 4 August 2021). The magenta parts showed the model predictions with the observational data for the period from 13 May to 30 June 2022

In view of model performance judged by DIC [33], the transmission model using Google mobility data had lower values of DIC than that using the Government response index (Additional file 1: Table S6) and thus was the one used to present the rest of the results. Further, the model variant that considered three turning points in ascertainment rate (ASC) during the study period, which was better than that with two turning points (Additional file 1: Table S6), was chosen for investigating the COVID-19 transmission dynamics in Laos. Our model well regenerated the outbreaks in Laos from 11 April 2021 to 12 May 2022 and the good agreement of model predictions with the data for the period from 13 May to 30 June 2022 further validated the model (Fig. 3).

### Estimation of model parameters

**Fig. 4:**
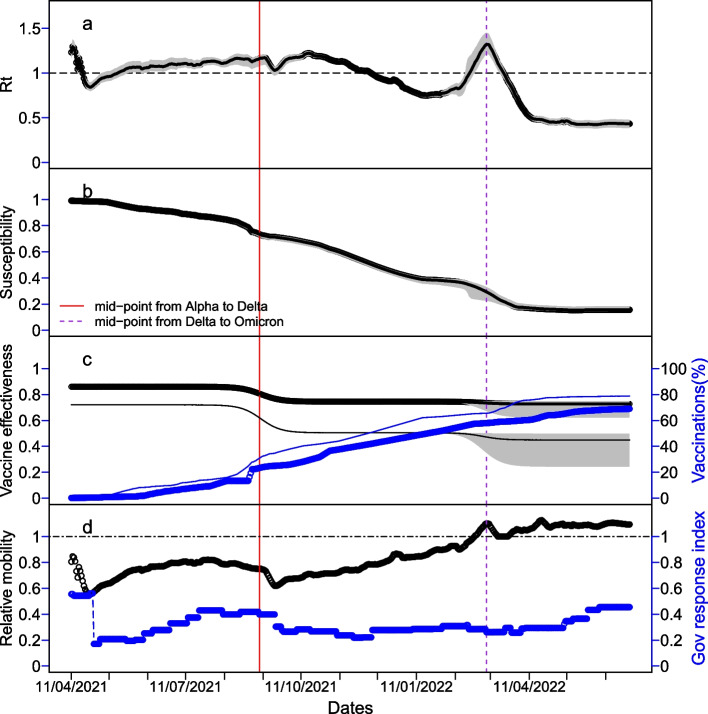
The temporal changes of key epidemiological characteristics during the period from 11 April 2021 to 30 June 2022 under the contact model via Google mobility. **a** time-varying reproduction number (*R*_t_), **b** susceptibility, **c** VE and accumulative proportion vaccinated (thin and bold lines represent those having at least one dose of vaccine and those fully vaccinated respectively), **d** relative contact change recorded in Google mobility data, and relative contact change due to government response Index. The red and purple vertical lines stood for the midpoints of transition from the Alpha to Delta variant and from Delta to Omicron variant of SARS-CoV-2 virus, respectively. In panels **a**–**c**, the thick black line represented median estimates and grey shading was their 95% credible intervals

Markov Chain Monte Carlo samplings indicated that among 25 model parameters to be estimated, five were restricted by prior assumptions while some other parameters such as the transmission coefficients for the different variants had tight uncertainty intervals (Table 1). Our modelling analysis showed that 2404 (95% credible interval (CrI): 1170–4354) people were infected on 11 April 2021 and the basic reproduction number of the Alpha variant was estimated to be 1.55 (95% CrI: 1.47–1.64) (Table 1); after reaching a peak one-half month later (26 April 2021), the effective reproduction number (*R*_t_) went down to 0.86 (Fig. 4a) and the outbreak decreased (Fig. 3b). This was most likely due to the substantial reduction in people’s mobility and the increase in government response index within a short period of time (Figs. 1a and 4d). As the control measures gradually relaxed and people’s mobility resumed, *R*_t_ increased and exceeded 1.0 around the middle of May 2021. This was followed by large daily numbers of imported cases from July 2021 and a slow increase in local cases, which continued up to early September 2021. Delta variant was first reported among the importation on 7 July 2021 [31]; our modelling analysis indicates that the fraction of infections with Delta exceeded 50% (i.e. Delta variant dominated) in Laos from 9 September 2021 (95% CrI: 6–16 September 2021), agreeing with the sequencing sample data (Fig. 1c). This was companied by a quick increase in local cases, an indicator of high transmissibility of the Delta over Alpha variant: Delta variant was estimated to be 1.88 (95% CrI: 1.77–2.01) times more transmissible than the Alpha variant (Table 1).

**Table 1:** Prior and posterior distributions for parameters of transmission model under Google mobility-driven contact rate along with the parameters fixed

Parameter	Prior	Posterior (95% CrI)	Unit/reference
Initial number of seeds (I_0_) on 11 April 2022	U [100, 6000]	2404.3 [1170.4, 4354.0]	–
Initial growth rate (ψ_r_)	U [0.02, 0.40]	0.059 [0.052, 0.068]	Day^−1^
Transmission coefficient of Alpha variant (B_α_)	–	0.44 [0.42, 0.47]	Contact^−1^ day^−1^
Basic reproduction number of Alpha variant (R_0,α_)	–	1.55 [1.47, 1.64]	–
Transmission coefficient of Delta variant (B_δ_)	U [0.50, 5.00]	0.83 [0.81, 0.87]	Contact^−1^ day^−1^
Transmission coefficient of Omicron variant (B_ο_)	U [0.80, 15.0]	2.77 [2.36, 3.13]	Contact^−1^ day^−1^
Midpoint in transition from Alpha to Delta variant (τ_α*_)	U [5 Jul, 13 Oct 2021]	09 [06, 16] Sep 2021	Days
Midpoint in transition from Delta to Omicron variant (τ_δ*_)	U [5 Feb, 1 Apr 2022]	5 Mar [20 Feb, 11 Mar] 2022	Days
Susceptibility of W to Delta variant (σ_δ_)	U [0.13, 0.70]	0.64 [0.47, 0.697]^#^	Hall et al. 2021[35]
Susceptibility of W to Omicron variant (σ_ο_)	U [0.13, 0.60]	0.136 [0.13, 0.165]^#^	
Relative infectivity for Alpha variant of the singly vaccinated (ε_1,α_)	Fixed ε_1,α_ = ε_2,α_	0.40	Assumed
Relative infectivity for Alpha variant of the fully vaccinated (ε_2,α_)	Fixed	0.40	Eyre et al. 2022 [36]
Relative infectivity for Delta variant of the singly vaccinated (ε_1,δ_)	Fixed ε_1,δ_ = ε_2,δ_	0.63	Assumed
Relative infectivity for Alpha variant of the fully vaccinated (ε_2,δ_)	Fixed	0.63	Eyre et al. 2022 [36]
Relative infectivity for Omicron variant of the singly vaccinated (ε_1,ο_)	ε_1,ο_ = ε_2,ο_	0.26 [0.25, 0.28]	Assumed
Relative infectivity for Omicron variant of the fully vaccinated (ε_2,ο_)	U [0.25,1.00]	0.26 [0.25, 0.28]^#^	Assumed
Ascertainment rate ASC1 (%)	U [1, 95]	13.20 [7.98, 25.04]	–
Turning point in ascertainment (τ_ASC1_)	U [10 May, 19 Jun 2021]	21 May [18, 25 May 2021]	Days
Ascertainment rate ASC2 (%)	U[1, 95]	1.81 [1.26, 2.40]	–
Turning point in ascertainment (τ_ASC2_)	U [20 Jun, 15 Dec 2021]	14 [11, 17] Sep 2021	Days
Ascertainment rate ASC3 (%)	U [1, 95]	5.15 [4.80, 5.66]	–
Turning point in ascertainment (τ_ASC3_)	U [1 Jan,5 Apr 2022]	14 [13, 16] Mar 2022	Days
Ascertainment rate ASC4 (%)	U [1, 95]	23.44 [17.17, 31.98]	–
CFR due to Alpha variant (%)^♦^	U [cfr0/4, 4 × cfr0]	0.38 [0.21, 0.60]	–
CFR due to Delta variant (%)^♦^	U [cfr0/4, 6 × cfr0]	1.05 [0.96, 1.15]	–
CFR due to Omicron variant (%)^♦^	U [cfr0/5, 4 × cfr0]	0.28 [0.18, 0.39]	–
Dispersion parameter for Cases (η_Case_)	U [5, 200]	32.14 [27.713, 37.218]	–
Dispersion parameter for deaths (η_Death_)	U [1.01, 5.00]	1.008 [1.005, 1.021]	–
VE of 1 dose against Alpha e_1,α_ (%)	Fixed	72.0	Hall et al. 2021 [4]
VE of full doses against Alpha e_2,α_ (%)	Fixed	86.0	
VEof 1 dose against Delta e_1,δ_ (%)	Fixed	50.5	Pouwels et al. 2021 [14]
VE of full doses against Delta e_2, δ_ (%)	Fixed	74.5	
VE of 1 dose against Omicron e_1,ο_ (%)	U [0.05, 0.50]	44.93 [24.32, 49.80]^#^	–
VE of full doses against Omicron e_2,ο_ (%)	U [0.10, 0.75]	72.72 [62.26, 74.90]^#^	–
Rate at which Delta replace Alpha variant (C_δ_)	U [0.05, 2.0]	0.071 [0.055, 0.104]	–
Rate at which Omicron replaces Delta variant (C_ο_)	U [0.05, 2.0]	0.127 [0.089, 1.636]	

^♦^cfr0 = 0.44% was the naïve estimate by dividing the total number of deaths by the total of cases reported

^#^Markov Chain Monte Carlo samplings indicated that these five parameters were restricted by prior assumptions

Laos started vaccination on 17 March 2021 and, up to May of 2022, and there were 79.6% of the population having at least one dose and 68.4% having fully vaccinated (Figs. 1b and 4c) [25]. The susceptibility around the end of 2021 was about 60% (Fig. 4b). This low level of susceptibility resulted from two aspects: the relatively high VE against the Delta variant: 50.5% for those having one dose, and 74.5% for those having fully vaccinated [14] (Fig. 4c), and the low risk of re-infection (64.2%) for those who have lost their immunity (Table 1). Working together with some slightly relaxing restrictions on contacts which still reduced the contact rate to less than 80% of the normality prior to the pandemic (Fig. 4d), immunity acquired via vaccination and infection successfully suppressed the transmission of the Delta variant, so *R*_t_ was brought down below 1.0.

However, the epidemic trend in Laos changed because of the importation of the Omicron variant. It was first reported among importation to Laos on 1st February 2022 [31], and our analysis indicated that Omicron dominated in Laos from 5 March 2022 (95% CrI: 22 February–11 March 2022) (Table 1). It further showed that VE against the Omicron variant slightly reduced to 44.9% (95% CrI: 24.3–49.8%) and 72.7% (95% CrI: 62.3–74.9%) for those having one dose and fully vaccinated, respectively. The relative infectiousness of vaccinated individuals was estimated to decrease from 63% for Delta to 26% (95% CrI: 25–28%) for Omicron, while those who had lost their immunity became less risk to be reinfected with Omicron than Delta (13.6% (95% CrI: 13.0–16.5%) versus 64.2% (95% CrI: 46.6–69.7%)). Furthermore, the Omicron variant was estimated to be 3.34 (95% CrI: 2.84–3.74) times more transmissible than the Delta variant (Table 1). These increased *R*_t_ beyond 1.0 and induced a sudden rise in the new local cases once Omicron variant was predominated from 5 March 2022 (Fig. 3). Nevertheless, the quick and large increase in infections also quickly accumulated the people that acquired immunity and thereafter reduced the overall susceptibility: It downgraded to < 30% from the middle of March 2022 (Fig. 4b) with *R*_t_ dropped to about 0.5 (Fig. 4a) and the new infections decreased quickly (Fig. 3a).

Delta variant appeared to be the severest variant among the three variants: its case fatality rate (CFR) was estimated to be 1.05% (95% CrI: 0.96–1.15%) while the estimates of CFR by the Alpha and Omicron variants were 0.38% (95% CrI: 0.21–0.60%) and 0.28% (95% CrI: 0.18–0.39%), respectively.

ASC of infection varied substantially over the outbreak period [34]. To model its temporal changes, it was simply assumed that ASC changed at three turning points. Model calibration suggested that it was 13.2% (95% CrI: 8.0–25.0%) from 11 April to 21 May 2021, decreased to 1.8% (95% CrI: 1.3–2.4%) from 22 May to 14 September 2021, increased to 5.1% (95% CrI: 4.8–5.7) from 15 September 2021 to 14 March 2022, and further increased to 23.4% (95% CrI: 17.2–32.0%) since 15 March 2022.

### Sensitivity analysis

Comparison [4, 10, 15] among the six vaccines used in Laos [25] showed that their VE was comparable with the two inactivated vaccines (Sinopharm and Sinovac) having low VE (Additional file 1: Table S4). In the above analysis, we fixed VE against Alpha and Delta variants at the estimates for Pfizer/BioNTech and Oxford/AstraZeneca vaccines, i.e. 72.0%, 86.0% against Alpha infection [4], and 50.5% and 74.5% against Delta infections [14] for those having one dose of vaccine and those having fully vaccinated, respectively. Model calibration showed that VE against the Omicron variant was 44.9% (95% CrI: 24.3–49.8%) and 72.7% (95% CrI: 62.3–74.9%) for those having one dose of vaccine and those having fully vaccines respectively, which were slightly lower than that against Delta variants and comparable with the empirical estimates [6, 7, 15]. To test how the variation in VE of the six vaccines affected our results, sensitivity analysis under six scenarios was performed by either increasing by 10% or decreasing by 15% the four VE in absolute value or assuming the longest or shortest duration of immunity from the estimates summarised in Additional file 1: Table S5. The estimates in Additional file 1: Table S7 indicated that the change in parameter estimates under five scenarios that altered VE and duration of vaccination was small. For example, the absolute values of relative changes in median estimates of transmission-related parameters such as initial growth rate (ψ_r_) and VE against the Omicron variant were smaller than 6%; the absolute values of relative changes in the three CFRs were smaller than 14%. Even though the absolute values of relative changes in median estimates of four ASCs could be 41%, the actual changes could be smaller in view of the wide uncertainties in these parameters.

Vaccination can also reduce transmissibility if the vaccinated gets infected by reducing viral load and shortening the duration of infectiousness [6]. It was found [36] that vaccine-associated reductions in the transmission of the Delta variant were smaller than those with the Alpha variant for the fully vaccinated (1–0.63 versus 1–0.40). If the vaccine-associated reductions in transmission for those having one dose of vaccine were equal to that for the fully vaccinated, our analysis shows that vaccination reduced transmissibility of the Omicron variant by 100–26% = 74% (Table 1). If assuming the transmissibility of the singly vaccinated was 30% more than those fully vaccinated, this generated an absolute value of relative changes in the estimates of all parameters being smaller than 5% (Additional file 1: Table S7). The sensitivity analysis suggested that our model inference results were robust.

### Impact of control measures and vaccination

**Fig. 5:**
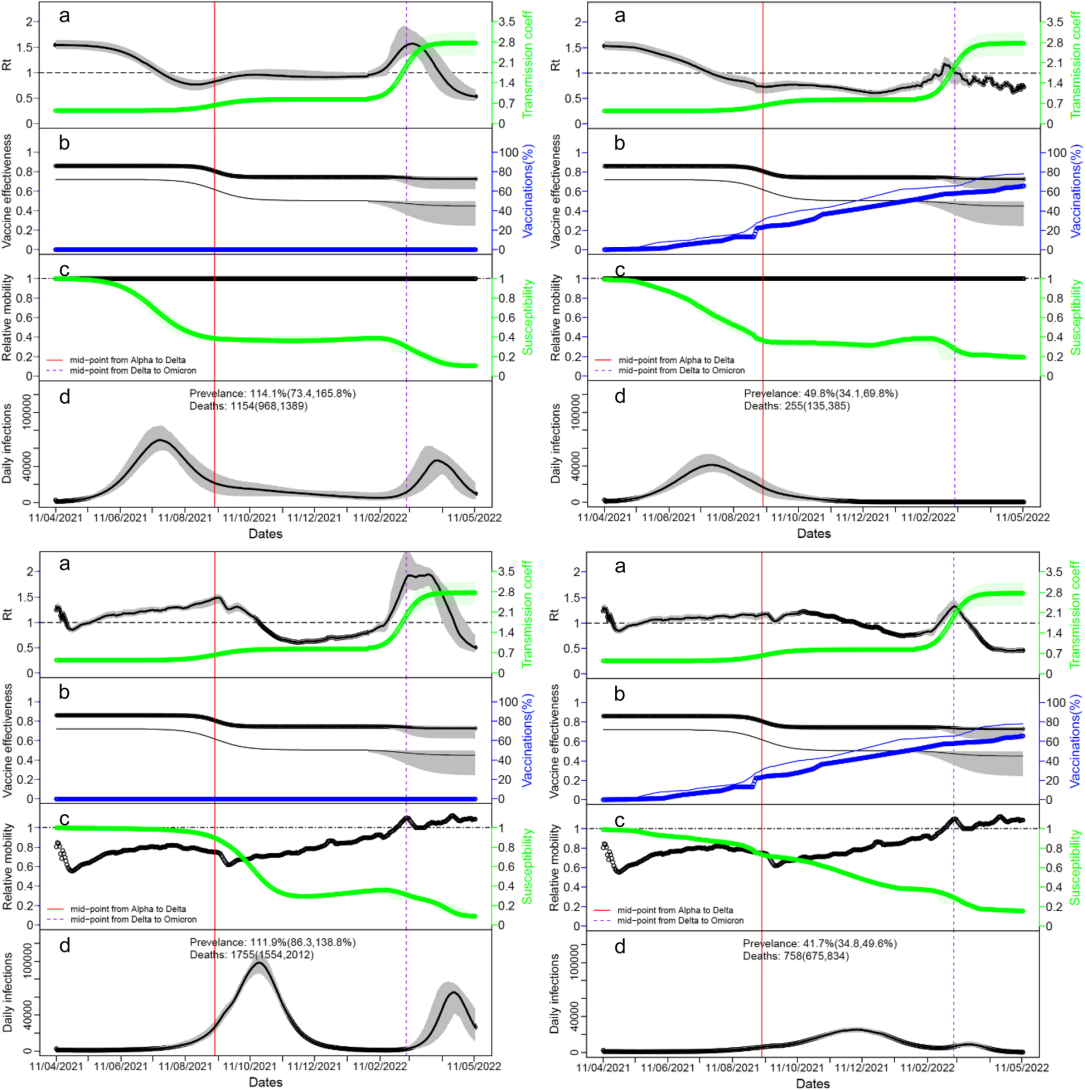
Impact of behaviour change and vaccinations on transmissibility, susceptibility, and outbreaks of SARS-CoV-2 infections in Laos. Four situations are considered: counterfactual situation without behaviour change and vaccination (top left); situation without reduction in contact rate induced by protective behaviours (i.e. No behaviour change) (top right); situation without vaccination (bottom left); the baseline situation for the actual epidemic outbreaks in Laos (bottom right). For each situation, the following characters were shown **a** time-vary reproduction number *R*_t_, **b** proportions of singly and fully vaccinated and VE; **c** reduction in contact rate and susceptibility to SARS-CoV-2 infection; **d** the predicted incidence from 11 April to 12 May 2022. In panel **b**, the thin and bold lines represented the values for the single and fully vaccinated respectively; in panel **d**, the prevalence of SARS-CoV-2 infection on 12 May 2022 and cumulated deaths were shown. In panels **a**, **b**, and **d**, the thick black line represented median estimates and grey shading was their 95% credible intervals

To assess the impact of control measures and vaccination, we considered a counterfactual situation where no behaviour change and no vaccines were delivered to control COVID-19 in Laos. In the counterfactual situation without behaviour change and vaccination, Laos people would experience two large outbreaks from May to September 2021 and from March to May 2022, and the whole population would be infected with some people being infected multiple times and 1154 (95% CrI: 968–1389) would die (Table 2; top left panel of Fig. 5). The first outbreak was due to the high susceptibility of people to Alpha variant, and the second due to the high transmissibility of the Omicron variant which was 6.25 (95% CrI: 5.22–7.36) times more transmissible than Alpha variant (Table 1).

**Table 2:** Projected impact of control measures for the transmission model with contact rate determined by Google mobility data of Laos. Projections for the outbreaks from 11 April 2021 to 12 May 2022 are included under 12 scenarios compared to the counterfactual scenario with no control measures

Conditions	Prevalence (%)	Deaths	Peak date of the largest wave	Peak size of the largest wave	% of infections (deaths) prevented compared to counterfactual
Counterfactual (no behaviour changes and vaccine)	114.1 [73.4, 165.8]	1154 [968, 1389]	2021–07-18	69,122 [57,749, 85,151]	–
With vaccine alone	49.8 [34.0, 69.8]	257 [139, 388]	2021–07-19	41,584 [33,372, 53,541]	56.6 [53.8, 58.5] (77.7 [64.8, 88.3])
With vaccine alone and early arrival of Delta and Omicron variants^*^	65.6 [48.2–84.3]	359 [230, 521]	2021–08-11	60,131 [42,366, 73,964]	43.0 [39.1–47.3] (68.5 [53.6-81.6])
With behaviour changes alone	111.9 [86.3, 138.8]	1752 [1558, 2005]	2021–10-20	98,725 [86,426, 108,114]	2.8 [1.1, 6.2] (− 52.5 [− 30.5, − 76.1])^#^
With both behaviour changes and vaccine (baseline)	41.7 [34.8, 49.6]	756 [684, 835]	2021–12-05	25,274 [22,621, 28,127]	63.8 [62.1, 66.1] (34.2 [22.1, 45.5])
With both behaviour changes and vaccine but Halving VE	58.3 [50.8, 66.4]	1056 [958, 1172]	2021–11-23	47,770 [43,536, 51,585]	49.5 [47.2, 51.6] (8.5 [24.5, − 10.2])^#^
With both behaviour changes and vaccine but VE = 100%	28.1 [20.9, 37.6]	507 [434, 589]	2021–12-08	11,622 [9,960, 13,405]	75.5 [72.9, 77.7] (56.0 [48.0, 63.7])
With both behaviour changes and vaccine but life-long immunity	34.2 [26.5, 48.9]	601 [513, 770]	2021–11-27	18,226 [15,990, 20,505]	70.1 [63.8, 73.0] (47.3 [33.2, 57.5])
With both behaviour changes and vaccine but VE = 100% over life-long	14.4 [9.8, 23.6]	218 [169, 317]	2021–11-13	5816 [4816, 6933]	87.3 [84.0, 88.8] (81.0 [73.2, 85.3])
No behaviour changes, with vaccine alone but Halving VE	59.8 [39.6, 84.8]	363 [198, 528]	2021–07-20	47,086 [37,924, 59,913]	47.8 [43.8, 51.8] (68.0 [52.3, 83.5])
No behaviour changes, with vaccine alone but VE = 100%	44.7 [31.4, 62.0]	202 [115, 309]	2021–07-21	38,850 [31,224, 50,088]	60.9 [58.8, 62.5] (82.4 [72.6, 90.3])
No behaviour changes, with vaccine alone but life-long immunity	47.2 [33.2, 65.8]	213 [124, 334]	2021–07-21	41,564 [33,794, 53,918]	58.6 [56.0, 60.4] (81.3 [70.6, 89.8])
No behaviour changes, with vaccine alone but VE = 100% over life-long	42.2 [30.2, 58.0]	174 [99, 274]	2021–07-21	38,291 [300,821, 48,863]	63.1 [60.3, 65.0] (85.2 [76.5, 91.5])

^*^To consider the possible effect of behaviour changes, we assumed in this scenario that without behaviour changes, Delta and Omicron variants arrived at Laos one month early

^#^the negative values represented the increase

The direct effects of control measures were related to the change in the transmission rate such as reproduction number *R*_t_, and their further consequence would be in avoided infections [37]. To compare the four scenarios, *R*_t_, susceptibility and daily infections were shown in Fig. 5. Owing to the consecutive replacement of variants along with the control measures, the temporal change in *R*_t_ and susceptibility was their combined results. With vaccination alone from March 2021, 49.8% (95% CrI: 34.0–69.8%) of Laos population would have been infected by the end of May 2022 and only 257 (95% CrI: 139–388) died (Table 2; top right panel of Fig. 5). In this counterfactual situation (i.e. without behaviour change), Laos population would experience only one large outbreak during the Alpha-dominated period from May to September 2021. The protection due to immunity of the large number of infections during the outbreak and the continued vaccinations could help avoid any outbreaks later even with the emergence of the highly transmissible Omicron variant. Compared to the counterfactual situation without control measures, vaccination alone was estimated to decrease infections and deaths by 56.6% (95% CrI: 53.8–58.5%) and 77.7% (95% CrI: 64.8–88.3%), respectively (Table 2).

By enforcing NPIs alone so people would reduce their contact rate through protective behaviours and restricting their mobility; this helped delay the first outbreak from the Alpha-dominated period to the Delta-dominated period from September to November 2021 (bottom left panel of Fig. 5). All the Laos people were very likely to get infected by the end of May 2022 and 1752 (95% CrI: 1545–2008) people died. With NPIs alone and their sequent behaviour change, the first outbreak was delayed but not flattened: the daily new infections on the peak were about 100 thousand while the peak size was about 70 thousand without control measures. Compared to the counterfactual situation without control measures, behaviour change partly induced by NPIs alone decreased the total infections only by 2.8% (95% CrI: 1.1–6.2%) but increased deaths by 52.5% (95% CrI: 30.5–76.1%) (Table 2). This worse outcome under NPIs alone than that without control measures was due to the emergence of the Delta variant that was 1.88 (95% CrI: 1.77–2.01) times more transmissible and 2.78 (95% CrI: 1.70–5.20) times more deadly than Alpha variant (Table 1). Anyway, this analysis may cast doubt on the extent of the benefit of mere restrictions to suppress transmission of the SARS-CoV-2 virus [38].

In a situation that includes both NPIs and vaccination together as what happened in Laos, 41.7% (95% CrI: 34.7, 49.6%) of the Laos population would have been infected by the end of May 2022 with 754 (95% CrI: 685–836) deaths (bottom right panel of Fig. 5). NPIs helped delay the first outbreak to the Delta-dominated period from October 2021 to January 2022, the protection induced by continued vaccination kept the outbreak at much low levels. Compared to the counterfactual situation without control measures, behaviour change, and vaccination together reduced 63.8% (95% CrI: 62.1–66.1%) of infections and 34.3% (95% CrI: 22.1–45.5%) of deaths (Table 2).

## Discussion

Since the emergence of COVID-19 in December 2019, control measures have been used to tackle the SARS-CoV-2 virus spread. Under the selection pressures of control measures and vaccination, SARS-CoV-2 has quickly evolved. The most compelling task was to understand how the COVID-19 pandemic has been defined by behaviour change, vaccination, and new variants. In this study, we used mathematical modelling and chose Laos as a case study to illustrate the underlying determinants for the COVID-19 evolution. Our modelling showed that the COVID-19 outbreak in Laos since April 2021 was shaped collectively by behaviour change, vaccination, and new variants when the sequential variants were becoming more transmissible and immune evadible. People’s responsive behaviours and vaccination limited the spread of infection, but the continuous emergence of more transmissible and immune escape variants challenged SARS-CoV-2 control.

Incorporating factors that influenced the SARS-CoV-2 spread in Laos, a transmission model was proposed to describe the three consecutive waves of COVID-19 outbreaks from 11 April 2021 to 12 May 2022. The basic reproduction number for the Alpha variant was estimated to be 1.55 (Table 1), comparable with other estimates [8, 9]. Delta and Omicron variants were 1.88 and 6.25 times more transmissible than Alpha variants, respectively, agreeing with other studies [13, 39]. Simulations indicated that it took about two months for Delta to replace the Alpha variant but only about 1 month for Omicron to replace the Delta variant in Laos (Table 1; Fig. 1c). The slow replacement of Alpha by the Delta variant in Laos was comparable to that in England [40]. The quick replacement of Delta by the Omicron variant was also observed in other countries [16, 41]. The different replacement speeds might result from the higher transmissibility and immunity evasion of new invader variants.

Our estimates of CFR showed that: CFR_Delta_ > CFR_Alpha_ and CFR_Delta_ > CFR_Omicron_. This relationship agrees with other studies [6, 17]: Lauring et al. [6] showed that the respective in-hospital mortality for the Alpha, Delta, and Omicron variants was 7.6%, 12.2% and 7.1%. Our estimate of the ratio of CFR_Omicron_ versus CFR_Delta_ was 0.27(95% CrI: 0.16–0.38), agreeing with 0·31 (95% CrI: 0·26–0·37) from England [17]. Overall, CFR in Laos was about 10 times smaller than others: for example, CFR_Alpha_ in Laos was estimated to be 0.38% while it was 5.3% in Turkey [42]. The low severity in Laos was likely to result from environmental and demographical factors [38].

ASC of infection is an important parameter but difficult to measure [34, 43]; it depends on factors such as the proportion of symptomatic infection and the method and scale of testing. In the early stage of the pandemic, ASC was estimated to range from 2.38 to 99.6% across countries [44]. It varied over time as medical resources and testing methods improved [43]: for example, ASC in China increased from 6% before 20 January 2020 to 25–55% after 20 January 2020. In this study, we found that ASC in Laos varied, and its temporal pattern reflected the control policies and capabilities of Laos’ health authorities (Additional file 1: Table S3). Before May 2021, enhanced tests were conducted on all close contacts of confirmed cases and the suspected groups. The limited testing capacity of Laos health authorities can cope with the low number of infections during the first wave and ASC was expected to be high (13.2%). After May 2021, the testing strategy was shifted to prioritising the symptomatic infections and payment for testing was required, which decreased ASC to a very low level (1.8%). Since September 2021, the installation of more PCR Laboratories and the use of antigen rapid diagnostic test improved the testing capacity, increasing ASC to 5.1%. In March 2022, Laos’ Ministry of Health has disseminated an electronic data collection form, so those self-testing positives were counted as confirmed COVID-19 cases, increasing ASC to a higher level (23.4%).

The temporal pattern in ASC was a key to explain the COVID-19 evolution in Laos. With the varying ASC, the peak daily number of infections during the Omicron-dominated period from March to May 2022 was much smaller than the Delta-dominated period from August 2021 to Feb 2022 (Fig. 3a); Although the infection pattern was in contrast to the observed data (Fig. 1a), it was consistent with the observation in Japan [33] where Delta variant caused the largest wave among the three variants. Up to 12 May 2022, 41.7% of the Laos’ people have been infected (Fig. 3; Table 2). Along with the 79.6% of the population vaccinated with at least one dose, the susceptibility to the Omicron variant was 20% in May 2022 (Fig. 4).

Increasing the level of immunity through vaccination is an effective measure to control vaccine-preventable infectious diseases [26]. During the period from April 2021 to May 2022, there were two stages in which herd immunity was established. Up to the middle of December 2021, susceptibility continued to decrease and *R*_t_ reduced to below 1.0. As people’s mobility continued to relax towards normality during the period (Figs. 1 and 4d), a herd immunity level was suggested to have been achieved with respect to the Delta variant. Without the importation of the Omicron variant, COVID-19 would have been under control in Laos from then. Up to April of 2022, susceptibility decreased to about 20% and *R*_t_ to about 0.5; the observation that people’s mobility has resumed to normality (Fig. 4d) indicated a herd immunity with respect to the Omicron variant was established. Without any new variants that are more transmissible and evade more immunity, it would be expected that the outbreak in Laos would be brought under control.

To disentangle the impact of each control measure, we simulated transmission processes by removing one or more control measures. Counterfactual analysis (Table 2; Fig. 5 and Additional file 1: Fig. S4) indicated that protective behaviours enhanced by NPIs helped delay outbreaks but might not necessarily be able to flatten the outbreak size under the circumstances of the continued emergence of higher transmissible variants. With the emergence of more transmissible and more lethal new variants, NPIs alone might induce more infections and mortality (Fig. 5; Table 2). In the long-term, behaviour changes enhanced by NPIs avoided the advantage of inhibitory competition between variants [45]: implementation of NPIs protected people from infection of the current variant but allowed the future variants to infect the unprotected; Without protective behaviour enhanced by NPIs, more people would be infected and cross-protection from these infections would help avoid large outbreaks with new future variants. It is important to note that we expect smaller epidemics than the two modelled counterfactuals without behaviour change because of the reduction due to protective changes in people’s behaviours of their own accord which were also reflected in Google mobility data. The volatile changes in people’s behaviours due to potential feedback mechanisms were reasonable in the absence of any NPIs during the long period of time under investigation. This should further alleviate the epidemics without NPIs. Therefore, the effectiveness of measures could be somewhat overestimated. The short-term benefit of behaviour changes enhanced by NPIs to avoid the overwhelmingly collapse of the health care systems and to buy time for developing pharmaceutical interventions may be affected by the uncertain nature of oncoming variants.

Nevertheless, vaccination was still a good choice to control COVID-19 even with immunity waning and escape of novel variants [46]. In the situation with vaccination alone but its VE against infection being half of the current values, only 59.8% (95% CrI: 39.6–84.8%) would be infected with 363 (95% CrI: 198–528) deaths. This outcome was better than that with behaviour change alone (Table 2). If all vaccines were perfectly against infection for life-long, then 42.2% (95% CrI: 30.2–58.0%) would be infected with 174 (95% CrI: 99–274) deaths. Further combining with behaviour change enhanced by NPIs, the life-long perfect vaccine would reduce the prevalence to 14.4% (95% CrI: 9.8–23.6%) with 218 (95% CrI: 169–317) deaths (Table 2; Additional file 1: Fig. S4). The differences between these two situations were caused by the complicated interactions of NPIs with the features of oncoming variants [47]. This indicated that combining vaccination with NPIs would be the best choice to suppress future variants.

Control measures such as NPIs could affect the arrival times of the new variants of the SARS-CoV-2 virus. For example, without restrictions on human movements, the new variants could emerge early in Laos. If two new variants came one month early (i.e. Delta emerged on 7th June 2021, and Omicron emerged on 1st January 2022), vaccination alone would allow 65.6% (95% Cri: 48.2–84.3%) of the Laos population to experience infection with 359 (95% CrI: 230–521) deaths up to May 2022 (Table 2). That is, compared to the situation that Delta and Omicron emerged on 7th July 2021 and 1st February 2022, the early arrival caused an additional 15.8% infections and more than 100 deaths. In view of this, NPIs and vaccination together could achieve a larger benefit than the above analyses that ignored the effect of control measures on the arrival time of new variants.

Many modelling studies investigated the outcomes under different control measures when multiple SARS-CoV-2 strains were circulated. Four studies [12, 45, 48, 49] investigated the determinants of the consecutive waves of COVID-19 by calibrating their models to the outbreak data. By separating viral from human social features, Barreiro et al. [12] applied a stochastic geographical model to COVID-19 waves in England and found that the new variants with higher transmissibility quickly became prevalent. Nevertheless, the underreporting of disease was not considered [12]. As in our model, Fierro et al. [48] included new variants by modifying susceptibility and transmissibility. Seasonal variation and prevalence-based awareness for protective behaviours were identified as the most relevant mechanisms for the COVID-19 evolution in Italy while new variants and mobility variation only had marginal effects. The latter part of their findings differed from that of [12] and ours, which might arise due to their assumption of permanent immunity and definitions of mortality and detection rate. As variation in environmental factors such as temperature in Laos over four seasons (ranging from 32 to 38 °C) was much smaller than that in Italy (ranging from 15 to 32 °C), the seasonal effect in Laos was expected to be weak.

By further including asymptomatic infections and the simultaneous spread of multiple variants, Layton and Sadria [45] found that in addition to infectivity, NPIs and vaccination, the prevalence and enhanced infectivity of asymptomatic infections also influenced the spread of COVID-19 in Ontario, Canada. The spread of the Delta variant depended not only on NPIs and vaccination, but also on the types of vaccines; vaccinated individuals were more likely to suffer vaccine breakthrough with Delta. This agreed with ours: Delta variant suffered a higher chance of vaccine breakthrough than the other two variants (Table 1). As in our study, simultaneous and rapid deployment of booster vaccines and NPIs were recommended as effective measures. Nonetheless, they [45] ignored the underreporting of disease and borrowed nearly all the values of model parameters from literature. Chapman et al. [49] used an age-structured multi-strain model to fit symptomatic cases, hospitalisations, and deaths over three consecutive major waves in French Polynesia. They observed that altering the timing of lockdowns during the first two waves had non-linear effects on overall incidence owing to the resulting effect on the accumulation of population immunity.

NPIs were widely used in tackling COVID-19 spread at its early stage when vaccines and drugs were not available. To provide reliable information to policy makers, many studies have assessed the effectiveness of NPIs [50]. Recommendations have been proposed to standardise the methodologies of NPI effectiveness analysis [37]; one recommendation was to exploit variation both over time and between populations to assess the effects of single NPI rather than a combination of multiple NPIs. Our present study used observational times series data from a single population to assess the effect of a combination of all NPIs plus voluntary behaviour change. To further consolidate and generalise our modelling results, it was needed to conduct the analyses on other populations to identify important sources of uncertainty in estimates of the impact of NPIs, vaccination and variants.

### Strengths and limitations

The strength of our study lay in the use of multiple data streams (vaccination, behaviours, government policy, sequencing, importation) and many estimates of epidemiological features borrowed from empirical studies. This guaranteed the estimates of the key parameters that were comparable with other studies as well as a reliable understanding of the underlying determinants of the outbreak in Laos. The model performance showed that people’s mobility-induced contact rate can reasonably reproduce long-term outbreaks (Additional file 1: Table S6; Fig. S3). This was the advantage over the studies [28, 29] that focused on short-term outbreaks with one SARS-CoV-2 variant.

Multi-strain models might be better to describe interactions between variants. As co-infection was rarely reported [12], we utilised a one-strain model to approximate the replacement of one variant by another through modulating epidemiological characteristics such as VE, duration of immunity, risk of re-infection, transmissibility, and severity [48]. Even with the simplicity, our modelling captured the characteristic changes within the three waves and obtained the estimates of the transmissibility and severity of the three variants in Laos. This encouraged us to extend the model to countries which suffered more waves with more variants such as the UK.

The weakness of our study was ignoring heterogeneities in epidemiological features and processes. For example, we assumed homogenous mixing among individuals by ignoring variation in age, geographical location, and strata of society [27–29]. As six vaccines were rolled out in Laos, we simply modelled the temporal change in VE by tracking predominant variants and ignoring details of their allocation. The delays from symptom onset to reporting and confirmation were taken from the estimates in China and assumed to remain unchanged, but they might vary over the long period under study because of the improved management system.

In our study, we explicitly considered vaccine protection against infection but only implicitly considered its effect on disease (i.e. infectiousness). The vaccination effect against disease and mortality can be included in Additional file 1: equations (S16) and (S17) by introducing different incubation periods and case fatality rates for the vaccinated. As the data on the vaccination status of deaths were not available, model calibration cannot identify the difference in mortality between the unvaccinated and the vaccinated. Hence case mortality rate might have been underestimated.

Another limitation of our study was only applying our modelling to a single population, and hence, it cannot assess how variation in behaviour, vaccination, and variants over populations [37] affected our key assumption that effective contact among individuals was directly proportional to the change in mobility relative to the level before the pandemic. Conducting the modelling across several populations to reduce the risk of confounding and other factors will be our next topic of study to justify or extend our causal assumption.

## Conclusions

In this study, we estimated the transmissibility and mortality of three variants of the SARS-CoV-2 virus in Laos and found that the Omicron variant was the most transmissible and the Delta variant was the most severe. Ascertainment was low and variable but had a big impact on the estimation of transmissibility and severity so a robust ASC estimate was important for constructing the COVID-19 evolution. Counterfactual analysis suggested that vaccination was still a major force to control COVID-19 even with immunity waning and escape due to new variants, and simultaneous implementation of NPIs and vaccination would achieve the best benefit in suppressing future COVID-19 outbreaks.

## Supplementary Information

Supplementary Material 1. It contains the following supplementary information: Data of cases and deaths, google mobility and government response index; Data of Laos’ control policies and test strategy and capacity; estimates of VE and duration of immunity induced by vaccination; technical details of model and methods; and sensitivity analyses.

## Data Availability

Data for this study (i.e. Google mobility data [21], Oxford COVID-19 Government response index[23], Vaccination [25], and outbreak data [30]) come from publicly available datasets. Code and collated data to recreate the main results are available via GitHub at https://github.com/wlx0871/laosCOVID19modelling [51].
