# Supplementary material for: Modelling COVID-19 transmission dynamics in Laos under non-pharmaceutical interventions, vaccination, and replacement of SARS-CoV-2 variants
Source: BMC Glob Public Health. 2024 Jun 17;2:38. doi: 10.1186/s44263-024-00069-y (PMC11622881; doi:10.1186/s44263-024-00069-y)
Supplement: Supplementary file 1 — Supplementary Material 1. It contains the following supplementary information: Data of cases and deaths, google mobility and government response index; Data of Laos’ control policies and test strategy and capacity; estimates of VE and duration of immunity induced by vaccination; technical details of model and methods; and sensitivity analyses. [file 44263_2024_69_MOESM1_ESM.docx]

**Additional File 1**

**Modelling COVID-19 transmission dynamics in Laos under Non-Pharmaceutical Interventions, vaccination, and replacement of variants of SARS-CoV-2 virus**

**Xu-Sheng Zhang ^1^, Hong Luo ^2^, Andre Charlett ^1^, Daniela DeAngelis ^1,3^, Wei Liu ^4^, Peter Vickerman ^5^, Mark Woolhouse ^6^, and Linxiong Wu ^4,7^**

^1^ Statistics, Modelling and Economics, Data, Analytics & Surveillance, UK Health Security Agency, UK

^2^ Education College, Yunnan University, Kunming, Yunnan, P. R. China

^3^ Medical Research Council Biostatistics Unit, University Forvie Site, Robinson Way, Cambridge, UK.

^4^ School of Public Health, Kunming Medical University, Kunming, Yunnan, P. R. China

^5^ Population Health Sciences, University of Bristol, Bristol, UK

^6^ Usher Institute, University of Edinburgh, Edinburgh, UK

^7^ Yunnan Provincial Key Laboratory of Public Health and Biosafety, Kunming, Yunnan, P.R. China

**Content**

Page

**Data** 3

Fig S1 Daily number of confirmed cases, Google mobility data and government response index in Laos 3

Table S1 The late and low occurrence of COVID-19 in Laos 4

Table S2 The timeline of Laos’ control policy 5

Table S3 The timeline related to test strategy and capacity 8

**Estimates of effectiveness and duration of immunity induced by vaccination** 10

Table S4 Effectiveness of vaccines against variants of SARS-CoV-2 10

Duration of immunity induced by vaccination 12

Table S5 Some estimates of effectiveness and duration of vaccine 13

**Technical details of Model and Methods** 16

Transmission model 16

Disease reporting 21

Inference model 22

Assessment of assumptions about ascertainment and contact mechanisms 23

Fig S3 Model estimation of infections and model fit 25

Table S6 Model comparison and selection among contact mechanisms and ascertainment assumptions 26

**Sensitivity analyses**  28

Table S7 Parameters of the model incorporating Google mobility under different assumptions of duration of immunity 30

Fig S4 Impact of NPIs and vaccinations on transmissibility and susceptibility and outbreaks of SARS-CoV-2 infections in Laos 32

**References** 33

**Data**

The data of daily number of imported cases and local cases, data of Google mobility, and government response index in Laos from 22 January 2020 to 30 June 2022 were shown in Fig S1. The timeline of the control policies implemented in Laos were listed in Table S2.


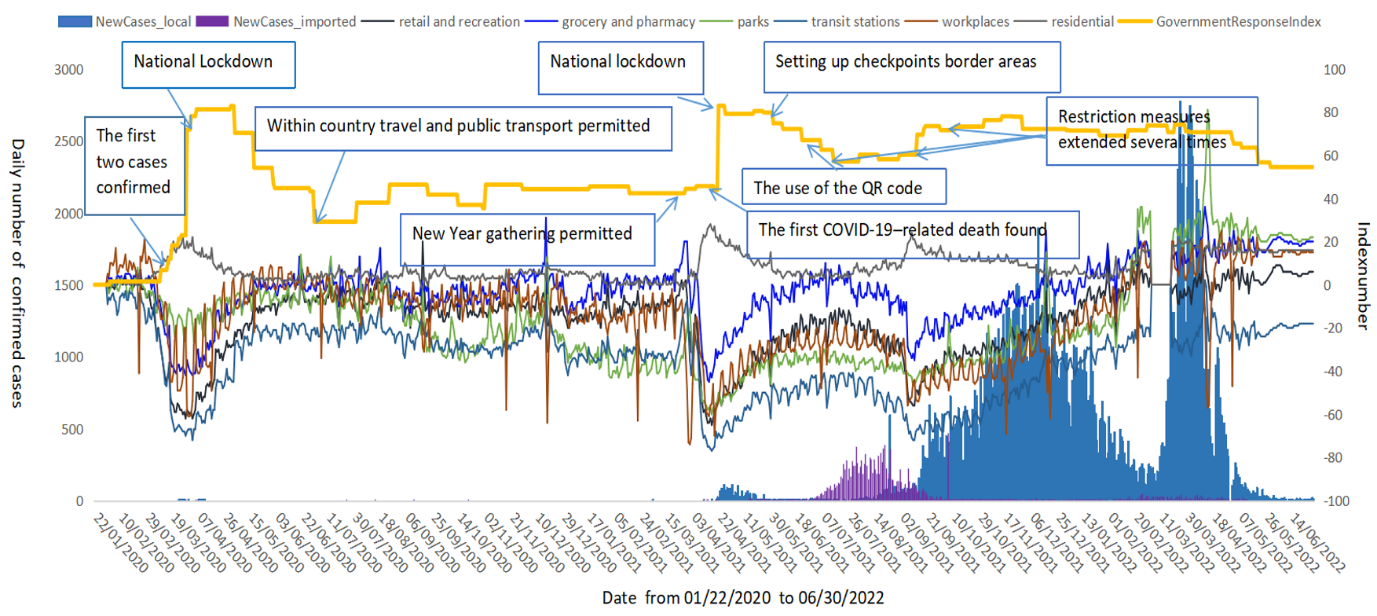


Fig S1 Daily number of confirmed cases, Google mobility data and government response index in Laos from 22 Jan 2020 to 30 June 2022. Google mobility data represented the daily numbers of visits at six locations: retail_and_recreation, grocery_and_pharmacy, parks, transit_stations, workplaces and residential [1]. Government response index was the combined index of government response indexes collected and provided by the Oxford COVID-19 Government Response Tracker (OxCGRT) [2].

**Table S1:** The late and low occurrence of COVID-19 in Laos comparing with its neighboring countries, USA, Brazil and Argentina

To illustrate the late occurrence of COVID-19 and its low burden in Laos, the report dates of first case(s) and death(s) due to COVID-19, the total number of cases and deaths up to two dates: 4 September 2020 and 14 April 2021, and the first report dates of three variants were listed in Table S1 among Laos and its neighboring countries, USA, Brazil and Argentina.

| Country | Report date of 1^st^ case(s) [3] | Report date of 1^st^ death(s) [3] | Total confirmed cases (deaths) up to 4/9/2020 [3] | Total confirmed cases (deaths) up to 14/4/2021 [3] | First report date of Alpha variant [4] | First report date of Delta variant [4] | First report date of Omicron variant [4] |
| --- | --- | --- | --- | --- | --- | --- | --- |
| Laos | 24/3/2020 | 9/5/2021 | 23 (0) | 52 (0) | 20/4/2021 | 7/7/2021 | 1/2/2022 |
| Vietnam | 23/1/2020 | 30/7/2020 | 1069 (35) | 2696 (35) | 2/1/2021 | 18/4/2021 | 19/1/2022 |
| Cambodia | 27/1/2020 | 11/3/2021 | 275 (0) | 4874 (35) | 3/2/2021 | 5/4/2021 | 15/12/2021 |
| Thailand | 22/1/2020 | 1/3/2020 | 3506 (59) | 35910 (97) | 3/1/2021 | 24/4/2021 | 6/12/2021 |
| Myanmar | 23/3/2020 | 31/3/2020 | 8515 (155) | 142605 (3206) | 1/4/2021 | 1/6/2021 | 29/12/2021 |
| China | 8/12/2019 | 22/1/2020 | 90187 (4634) | 99022 (4636) | 31/12/2020 | 24/4/2021 | 9/12/2021 |
| USA | 22/1/2020 | 29/2/2020 | 6997165 (201784) | 31523628 (560426) | 27/11/2020 | 23/2/2021 | 1/12/2021 |
| Brazil | 26/2/2020 | 17/3/2020 | 4663930 (139964) | 13688024 (362444) | 31/12/2020 | 20/5/2021 | 30/11/2021 |
| Argentina | 3/3/2020 | 8/3/2020 | 678266 (14766) | 2604157 (58542) | 16/1/2021 | 24/4/2021 | 6/12/2021 |

**Table S2:** The timeline of COVID-19 outbreaks and control measures implemented in Laos [5,6]

| Date | Control measures policy |
| --- | --- |
| 01/04/2020 | National lockdown: (a) Schools remain closed, (b) The Taskforce Committee for COVID-19 Epidemic Prevention, Control and Response is monitoring the latest developments and coordinates the authorities’ response. |
| 04/05/2020 | (a) The newly loosened measures allow the reopening of public offices and some private sector businesses and is expected to last until 17 May 2020; (b) Restaurants, hotels, resorts, guesthouses, coffee shops, barbershops, beauty salons as well as wholesale and retail outlets can resume their businesses, however, large-scale manufacturing plants and investment projects with large number of employees must abide by strict preventative measures introduced by the National Taskforce; (c) travel and public transport within each province are now permitted but inter-provincial travel and public transport including air transport are not. |
| 18/05/2020 | (a) Some classes at primary, and lower and upper secondary schools can resume on May 18 while all others are slated to open on June 2; (b) All indoor and outdoor sporting activities are now permitted; (c) The newly loosened measures allow public offices and businesses to resume and are expected to last until 1 June 2020; (d) Restaurants, food vendors, retail and wholesale outlets, fresh markets, supermarkets, personal care services as well as development projects, companies and factories are also permitted to resume operations, but must abide by strict preventative measures introduced by the National Taskforce; (e) Border checkpoints, night markets, and entertainment venues remain closed; (f) price control of essential goods is still in place; and (g) country travel and public transport are now permitted. |
| 10/06/2020 | (a) Large gatherings, including traditional ceremonies and celebrations, are now allowed; (b) The government announced that the last patient has fully recovered and discharged from hospital; (c) Foreign businessmen, investors, workers for large investment projects as well as diplomats and foreign experts with proper medical certification and authorization can enter the country but have to be quarantined for 14 days; (d) In-country travel and public transport are fully operational; (e) With the exception of certain checkpoints allowed by the government, border checkpoints for individuals and transportation of goods, will remain closed as will all international borders. |
| 01-31/08/2020 | (a) Prevention measures will be continuously imposed like closure of entertainment venues, karaoke, and gaming shops; All schools have resumed but must ensure hygiene and distancing practices; (b) All indoor and outdoor sporting activities are now permitted and audiences are allowed, however, entertainment venues remain closed; (c) Foreign diplomats, the staff of international organizations, experts, investors, business people, technical personnel, and workers deemed essential to take up missions in Lao PDR can enter the country upon approval by the task force committee; (d) Shuttle flights will be suspended; (e) Closure of traditional and local border checkpoints, except those permitted by the government for goods transport. International checkpoints remain closed for regular travellers, except essential crossings for Lao and foreign nationals who are permitted by the task force committee. Transportation of goods via the international checkpoints is allowed as normal; and (f) Suspending the issuing of tourist or visiting visas for those travelling from or transiting via countries where there is COVID-19 outbreak. |
| 01/10/2020 | The government has extended its COVID-19 prevention measures until October 31; Ban on charter flights will be eased; |
| 03/02/2021 | The Ministry of National Defence and the Ministry of Public Security, in cooperation with authorities in Vientiane and the provinces, especially border provinces, have been urged to strengthen border patrols until 28 February 2021 |
| 05/04/2021 | The government extended its COVID-19 prevention measures while also allowing people in Lao PDR to celebrate Lao New Year (Pi Mai Lao) on 14-16 April 2021 |
| 22/04/2021 | The Ministry of Education and Sports issued a notice ordering all public and private educational facilities from preschools to universities to be closed across Vientiane Capital |
| 04/05/2021 | The National Taskforce extended the lockdown period until 20 May 2021 |
| 13/05/2021 | The authorities in central and southern provinces that share borders with Thailand are taking action to prevent the spread of COVID-19 by setting up more checkpoints and patrolling border areas to prevent more imported cases of the virus. |
| 04/062021 | Education authorities have cancelled end-of-year exams. Under the new ruling, primary school students in grades 1, 2, 3 and 4 and secondary school students in grades 1, 2, 3, 5 and 6 who scored an average of 5 or more in all subjects (as assessed five months ago) will automatically enter a higher grade next academic year |
| 04/06/2021 | (a) The National Taskforce for COVID-19 Prevention and Control has announced that people living in the Lao PDR will be required to install the LaoKYC app on their smartphone and check in with a QR code as part of new contact tracing regulations. The use of the QR code will allow health authorities to monitor the movement of the population, making it easier to undertake contact tracing in the event of a COVID-19 localized outbreak; |
| 05/072021 | The government has decided to extend a Prime Minister’s Order designed to control the COVID-19 outbreak for another 15 days, amid growing concerns over the surge in infections in neighbouring countries |
| 19/08/2021 | Lao PDR extended COVID-19 restriction measures until 2 September 2021 |
| 09/09/2021 | The capital’s Taskforce for COVID-19 Prevention and Control recently issued a notice announcing the fines, which will range from LAK0.5 to LAK10 million depending on the severity of the offense |
| 19/09/2021 | Vientiane authorities ordered a full lockdown of the capital. Travel is banned on roads in the capital from 10 PM to 5 AM. Exceptions will be made for essential trips |
| 20/09/2021 | The Vientiane Capital Department of Industry and Commerce has issued a notice ordering the temporary closure of a number of retail businesses including supermarkets and minimarts on 20-30 September 2021.Meanwhile, fresh markets and petrol stations may open from 6 AM to 6 PM. The sale of alcohol at any location is strictly prohibited during this time. Failure to comply with COVID-19 prevention measures and lockdown regulations will be fined up to LAK10 million and face permanent closure; |
| 15/10/2021 | The Government of Lao PDR has extended COVID-19 restriction measures for another 15 days until 30 October 2021 |
| 31/10/2021 | The Government of Laos has extended COVID-19 restriction measures until 14 November 2021 and has included provisions for the reopening of schools; Entertainment venues remain closed. |
| 03/02/2022 | COVID-19 measures were relaxed |
| 01/03/2022 | Entry-exit Laos authorization requested |
| 18/03/2022 | The Ministry of Health is considering cancelling Lao New Year festivities in April because of the Omicron outbreak, with the daily number of new cases having risen from under 200 to upwards of 800. |
| 12/04/2022 | For any meeting with more than 100 participants, each attendee must perform an Antigen Rapid Diagnostic Test (AgRDT) test within 24 hours prior to attending the meeting. The Ministry of Industry and Commerce to guide and follow up with restaurants and stores to check the vaccine ID or vaccine certificate of each customer before entering. Passengers who use public transportation, such as airplane, bus or train, must show their vaccine certificate |
| 10/05/2022 | Open all international borders for Lao citizens and foreigners. Allow citizens in the countries that possess a visa exemption with Lao PDR to enter Lao PDR without applying for a visa. Allow visa applications at Lao embassies and consulate offices, or through E-Visa application online, or Visa on Arrival if applicable. - Unvaccinated or not fully vaccinated individuals above 12 years old need to undergo an Rapid Diagnostic Test (RDT) 48 hours prior to departure. No testing upon entry at all borders. Testing prior to departure as well as upon arrival is not required for those who are fully vaccinated.- Foreigners who enter Lao PDR and subsequently test positive for COVID-19 are responsible for all costs of treatment and isolation as advised by the Ministry of Health. Allow all types of vehicles to enter and exit Lao PDR as it would normally be prior to the COVID-19 pandemic. Entertainment venues and karaoke are allowed to open with COVID-19 safe measures. This notice is in force from 9 May. |
| 24/05/2022 | Individuals, entities, and non-Lao citizens must fill the form for requesting a certificate of vaccinations by themselves. Submit the form and attach the green card and copied passport on Monday, Wednesday and Friday at Vientiane Health Department. Tuesday, Thursday and Saturday are set to be certificate issuing days. The service will start from 24 May 2022. People can still request a certificate of vaccinations at Mother and Child Health Center (MCHC) from Monday to Friday. |
| 07/06/2022 | Appoint the taskforce for resolving the fuel shortage, product price, and foreign currency issues to: Ensure the regular importation and controlled price of fuel to meet demand. Introduce measures to supply foreign currency, foreign exchange management, and stabilize the Forex situation in private exchange shops and in the market. - Introduce and monitor strict measures for those who violate the law and harm the country's economy.- Control the price of goods in the market. In accordance with the government’s guidance to resolve the economic-financial crisis, from 1 June 2022 any payment for vehicles, including buying and selling, will be transferred through the bank system only. |

**Table S3:** The timeline related to test strategy and capacity in Laos [6]

| date | Test information about capacity and strategy |
| --- | --- |
| 02/03/2021 | Close contacts of two imported cases tested (close contacts were identified from persons in the same flight, persons in same vehicle from the Point of Entry (POE) to the quarantine facility or persons that stayed in the same room as the case in the quarantine facility) |
| 16/03/2021 | 13 out of 18 provinces are already implementing enhanced testing, with over 800 tests conducted among healthcare workers, and POE and quarantine staff since January and have not found positive cases to date. |
| 30/03/2021 | 15 out of 18 provinces are already implementing enhanced testing, with over 1,000 tests  conducted among healthcare workers, and POE and quarantine staff |
| 27/04/2021 | Enhanced testing in response to the current outbreak has led to detection of more cases with suspected exposure in high-risk locations. Although testing criteria should include testing all suspected cases, including severe acute respiratory infection (SARI) cases, the coverage is still being improved. There is also a possibility of undetected cases and wider transmission than is currently detected, as current testing strategies would still miss out on asymptomatic cases with no links to confirmed cases and identified high-risk locations. |
| 11/05/2021 | In Bokeo, 188 cases were reported in the last 7 days, which is an increase compared to 104 cases in the previous week. The majority of these cases are reported from the Special Economic Zone (SEZ) |
| 25/05/2021 | In Bokeo Special Economic Zone (SEZ), there is a recent decrease in the daily number of tests conducted as the testing strategy has been shifted to prioritize testing those that are symptomatic. Payment for testing is now required, which is expected to have resulted to a lower number of tests |
| 08/06/2021 | A stage-based surveillance and testing strategy has been drafted with the support of WHO. MoH is discussing with the Ministry of Public Security about the surveillance strategy and measures to detect and suppress and contain transmission in prisons, with WHO providing support. There are plans to expand the enhanced detection strategy in all provinces to include other high-risk occupations specific to each area (e.g., factory workers, market vendors, etc.) |
| 22/06/2021 | Enhanced detection in 13 markets and 11 factories in Vientiane Capital during 28 May to 22 June (n=9,910) did not find any positive cases. In other provinces, Covid-19 testing capacities of provinces are being increased using mobile PCR and GeneXpert. Twelve GeneXpert machines procured by WHO arrived this week and will be distributed to provinces. The number of tests per province in the last 14 days excluding tests among returnees, ranged from 10 - 1,300. |
| 06/07/2021 | The risk of undetected transmission in the wider community remains high. Increasing laboratory testing capacity through establishment of regional molecular laboratories. This will increase both the testing capacity and shorten the time for results to be available. |
| 20/07/2021 | Positivity rates among returnees were highest in Champasack (20% of 2,973 tests) and Savannakhet (19% of 3,201). There are no clear signals of community transmission in these 17 provinces, but the risk of undetected. |
| 03/08/2021 | Since 28 May to 3 August, over 9,700 tests were conducted as part of enhanced detection in 22 markets, 18 factories, and three companies, all tested negative. Over 500 samples were collected from seven prisons and detention centres since 22 June, all tested negative. There are no clear signals of community transmission in these 17 provinces, but the risk of undetected transmission in the wider community remains high. |
| 17/08/2021 | The number of community tests conducted in other provinces in the last 2 weeks ranged from 13-1,659 tests, with the positivity rates highest in Bokeo and Khammuane. Enhanced detection among frontline workers and high-risk settings continue to be conducted, with nearly 1400 samples collected in 9 provinces in August, including healthcare workers (n=370), POE staff (n=252), quarantine facility staff (n=271), markets (n=501), and factory workers (n=25). The National Centre for Laboratory and Epidemiology (NCLE) and WHO are supporting Khammuane province in strengthening testing capacity by setting up an rRT-PCR laboratory, technical support and equipment and supplies have been provided. |
| 31/08/2021 | Up to now, there are 10 provinces with PCR testing capacity. The maximum daily testing capacity at the provincial level is up to 500 samples. |
| 14/09/2021 | Currently WHO has been working for PCR installation and on-site training on molecular testing for SARS-CoV-2 in Luangprabang, Khammouane and Saravane provincial laboratory. To date, 10 provinces have PCR machines with the maximum testing at provincial level up to 500 samples per day. |
| 26/10/2021 | Training for the use of Antigen Rapid Diagnostic Test (RDT) was conducted to central hospitals on 26 October. Regional virtual trainings for all provinces are also scheduled this week. Training materials including videos have been developed with the support of WHO.  On 20 October, the Food and Drug Department (FDD) released a notice for all import-export companies for drugs and medical products in Lao PDR on the authorization of the importation of Antigen Rapid Diagnostic Tests (AgRDT) to be used for COVID-19 testing in Lao PDR. |
| 09/11/2021 | Training for the use of Antigen Rapid Diagnostic Test (RDT) was conducted for Vientiane Capital districts on 3 November 2021. Improving public understanding in the public and adherence of the appropriate use of AgRDT test kits as per the policy issued by MOH.  Ensuring frontline workers have the training and equipment and supplies required to protect  themselves to prevent infection |
| 23/11/2021 | NCLE is planning to update the case definitions for confirmed and suspected Covid-19 cases considering ongoing community transmission in many provinces and current use of Antigen Rapid Diagnostic Test (RDT). A meeting was held between NCLE and WHO and a follow-up meeting with provinces will be held prior to finalization of revised case definitions. |
| 07/12/2021 | Healthcare facilities are nearing capacity and need to be expanded, with care pathways and referral process clear and implemented well to ensure that severe cases can be appropriately managed to prevent deaths. Nationally, the number of tests conducted has decreased in the past two weeks, and testing of suspected cases including SARI and enhanced detection efforts in all provinces are being improved. |
| 19/01/2022 | Nationally, the testing of suspected cases including SARI and active case detection are being  implemented in all provinces, but there are remaining gaps and new clusters may still be missed. Ensuring that individuals that are tested positive by AgRDT are captured into the health system for monitoring purposes to prevent deaths in the community and improving public understanding and adherence on the appropriate use of AgRDT test kits as per the policy issued by MOH. |
| 02/02/2022 | Nationally, the testing of suspected cases including SARI and active case detection are being implemented in all provinces. |
| 01/03/2022 | Since 21 February, NCLE is setting up the rRT-PCR laboratory for SARS-CoV-2 testing at Sekong province, with support from WHO. On-site support will take up to 6 weeks, including setting-up the laboratory, providing lectures, and hands-on training on working station. |
| 29/03/2022 | The Ministry of Health has published and disseminated an electronic data collection form that can be completed by persons who self-tested as positive using AgRDTs so that they can be referred for case investigation and monitoring and transferred to a healthcare facility if clinically required. Those who report positive results from self-testing will be counted and managed as confirmed COVID-19 cases. WHO provided technical support in developing the form and managing the database. Nationally, the testing of suspected cases including SARI and active case detection are being implemented in all provinces, but there are gaps remaining and there is a risk that new clusters may be missed. There is widespread use of self-testing using antigen rapid testing kits, and there are ongoing efforts to encourage self-reporting of positive results from the rapid tests so that the reported case numbers reflect the true COVID-19 situation. |
| 12/04/2022 | The National Center for Laboratory and Epidemiology (NCLE), in conjunction with Department of Communicable Disease Control (DCDC) and Department of healthcare and Rehabilitation (DHR), and with WHO support, continue to hold meetings with provinces twice per week to discuss case investigation, enhanced surveillance, and any challenges related to the response. Persons who self-tested as positive using AgRDTs can self-report using an electronic form and are recorded as confirmed COVID-19 cases. WHO provided technical support in developing the self -report form and managing the database. |
| 24/05/2022 | The Ministry of Health (MoH) has developed a mobile application for home-based care monitoring with support from the e-government centre. They will conduct training for specialists in infectious diseases who will provide medical advice through this application. WHO is currently working with DHR to support further enhancement of oxygen treatment capacity in Vientiane Capital and all remaining 17 provinces, in close collaboration with Clinton Health Access Initiative (CHAI). This includes supporting the installation of three oxygen plants, and the procurement of additional High Flow Nasal Oxygen devices and BiPAP machines |

**Estimates of effectiveness and duration of immunity induced by vaccination**

**Table S4:** Effectiveness of vaccines against three variants of SARS-CoV-2.

Six different vaccines [6] were rolled out in Laos during the outbreak: Johnson & Johnson (a viral vector vaccine, also named as Janssen, Jcovden, Ad26.COV2.S), Pfizer/BioNTech (a mRNA vaccine, also named as Comirnaty, BNT162b2), Oxford/AstraZeneca (a viral vector vaccine, also named as Covishield, Vaxzevria, ChAdOx1), Sinopharm/Beijing (an inactivated vaccine, also named as Sinopharm BIBP, BBIBP-CorV, Sinopharm COVID-19, BIBP), Sinovac (an inactivated vaccine, also named as CoronaVac), Sputnik Light, and Sputnik V(a viral vector vaccine, also named as Gam-COVID-Vac). The vaccine effectiveness (VE) of inactivated vaccine (Sinopharm/Beijing, and Sinovac) were comparable with others’ but appeared weak [7,8,9]. Some estimates were summarised in Table S4, which showed large uncertainties in the estimates of VE against different variants of SARS-CoV-2 virus.

| Vaccine | VE of one dose of vaccine | | |
| --- | --- | --- | --- |
|  | Alpha variant | Delta variant | Omicron variant |
| Pfizer-BioNTech | 72% (58–86%) [10] | 45.2%(43.3–47.1%) [11]; 58%(51–63%) [12] | 42.8%(40.3–45.1%) [11]  16.5%(-19.5–41.6%) [13]  39.9%(24.8–52.3%) [14] |
| AstraZeneca |  | 42.9%(39.8–45.9%) [11]  43%(31–52%) [12] | 17.7%(14.3–21.0%) [11] |
| Johnson &Johnson | 74.2% (64.9–81.6%) [15]  76% (75–77%) [16] | 74%(71–77%) [16] |  |
| Sinovac |  | 15.5%(14.2–16.8%) [17] | -1.6%(-39.8–26.2%) [13]  32.7%(14.4–47.6%) [14] |
| Overall mean | 74.1% (range:72–76%) | 46.4% (range:15.5–74%) | 24.9%(range:0–42.8%) |
|  | VE of two doses of vaccine | | |
|  | Alpha | Delta | Omicron |
| Pfizer-BioNTech | 83.3% (95%CI 82.6–83.9%) [18]  86% (76–97%) [10] | 76.6% (95%CI: 76.0–77.0%) [19]  82%(79–85%) [12]  90.9%(89.6–92.0%) [11] | 65.5%(63.9–67.0%) [11]  40.0% (38.6–41.3%) [20] 27.6%(-6.3–20.1%) [13]  35.1%(26.6–42.5%) [14] |
| AstraZeneca | 71.5% (95%CI 69.2–73.6%) | 75.8% (95%CI: 75.2–76.3%)[19]  67%(62–71%) [12]  82.8%(74.5–88.4%) [11] | 48.9% (39.2–57.1%) [11] |
| Johnson &Johnson | – | 72.8% (95%CI: 71.8–73.6%) [19] |  |
| Sputnik-V | 85.7% (95%CI 84.3–86.9%) [18] | 71.8% (95%CI: 71.2–72.3%) [19] |  |
| Sinopharm | 68.7% (95%CI 67.2–70.1%) [18] |  |  |
| Sinovac | – | 64.0% (95%CI: 63.4–74.5%) [19]  65.9% (65.2–66.6%)[17]  39%(20–64%) [21] | 22.7%(-15.2–48.2%) [13] (BA.2)  25.1%(14.7–34.3%) [14] |
| Overall mean | 79.0% (range: 68.7–86.0%) median=83.3% | 71.7%(39–90.9%) median=72.8% | 37.8% (range:22.7–65.5%) median=35.1% |
|  | VE of three doses of vaccine | | |
| Pfizer-BioNTech |  |  | 41.4(23.3–55.2%) [13]  73.5%(66.6–79.2%)[14] |
| Sinovac |  |  | 32.4%(9–49.8%)[13]  51.0%(39.6–60.4%)[14] |
| Overall mean |  |  | 49.6% (range: 32.4–73.5%)  Median=46.2% |

**Duration of immunity induced by vaccination**

Vaccine surveys showed that the protection induced by vaccination against infection of SARS-CoV-2 waned quickly [22]. To estimate the average duration of immunity, we assumed it followed an exponential function as

$$VE\left( t \right)=VE_{0}exp(-\frac{t-t_{0}}{L_{imm}})$$

Here VE_0_ was the value of VE at time *t*_0_ and *L*_imm_ represented the mean duration of immunity. We used maximum likelihood method to obtain its estimate. Fig S2 showed the model fit and the estimate of durations of protection induced by A) one, B) two and C) three doses of vaccine BNT162b2 against Omicron variant of SARS-CoV-2 using data from [11]_._ Some estimates of immunity duration were lists in Table S5.


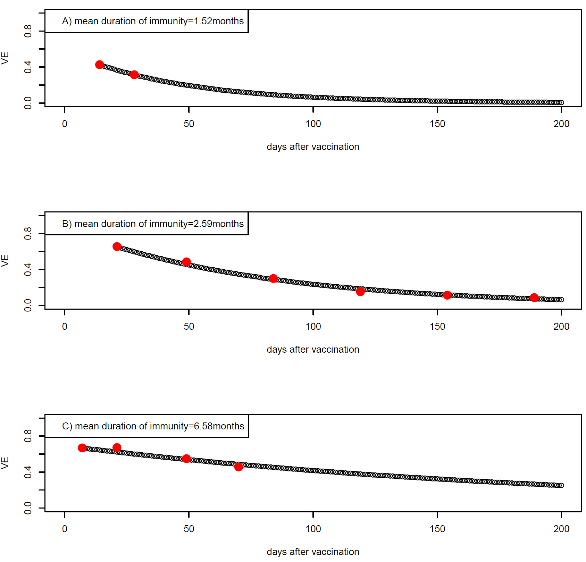


Fig S2 Estimation of waning rate in VE of A) one, B) two and C) three doses of BNT162b6 vaccine.

**Table S5:** Some estimates of VE and duration of vaccine against infections of SARS-CoV-2 virus

| Variant | Alpha |  | Delta | | | Omicron | | | Reference |
| --- | --- | --- | --- | --- | --- | --- | --- | --- | --- |
| Fully vaccinated (two doses) | | | | | | | | | Pouwels et al 2021 [12] |
| vaccine |  |  | BNT162b2 | mRNA_1272 | ChAdOx1 | BNT162b2 | mRNA_1273 | ChAdOx1 |  |
| Initial VE |  |  | 85% |  | 68% |  |  |  |  |
| duration |  |  | 10.53 months |  | 12.50 months |  |  |  |  |
| Average |  |  | VE_0_=76.5%, *L*_2,δ_  =11.43 months | | |  |  |  |  |
| Vaccine |  |  | BNT162b2 (2 doses) | Janssen (1 dose) |  |  |  |  | Cohn et al 2021[23] |
| Initial VE |  |  | 87% | 86% |  |  |  |  |  |
| Mean duration |  |  | 11.36 months | 5.92 months |  |  |  |  |  |
| Average |  |  | VE_0_=86.5%, *L*_2,δ_ =7.78 months | | |  |  |  |  |
| Vaccine |  |  | BNT162b2 | CoronaVac |  |  |  |  | Suah 2022 [24] |
| Initial VE |  |  | 90.8% | 74.5% |  |  |  |  |  |
| Mean duration |  |  | 18.52 months | 2.77 months |  |  |  |  |  |
| Average |  |  | VE_0_=82.7%, *L*_2,δ_ =4.82 months | | |  |  |  |  |
| Vaccine | BNT162b2 |  | BNT162b2 |  |  |  |  |  | Tartof et al 2021 [25] |
| Initial VE | 97% |  | 93% |  |  |  |  |  |  |
| Mean duration | 6.71 months |  | 5.35 months |  |  |  |  |  |  |
| **Average** | VE_0_=97%, *L*_2,α_ =6.71months | | VE_0_=93%, *L*_2,δ_ =5.35 months | | |  |  |  |  |
| One dose |  |  |  |  |  |  |  |  | Andrews et al 2022 [11] |
| Vaccine |  |  | BNT162b2 | mRNA_1272 | ChAdOx1 | BNT162b2 | mRNA_1273 | ChAdOx1 |  |
| Initial VE |  |  | 45.2% | 60.1% | 42.9% | 42.8% | 47.9% | 17.7% |  |
| Mean duration |  |  |  | 10.20 months |  | 1.52 months | 1.30 months |  |  |
| Average |  |  | VE_0_=53.9%, *L*_1,δ_ =10.20 months | | | VE_0_=36.1%, *L*_1,ο_ =1.40 months | | |  |
| Two doses |  |  |  |  |  |  |  |  |  |
| Vaccine |  |  | BNT162b2 | mRNA_1272 | ChAdOx1 | BNT162b2 | mRNA_1273 | ChAdOx1 |  |
| Initial VE |  |  | 90.9% | 94.5% | 82.8% | 65.5% | 75.1% | 48.9% |  |
| Mean duration |  |  | 15.15 months | 26.32 months | 8.33 months | 2.69 months | 3.13 months | 2.79 months |  |
| Average |  |  | VE_0_=89.4%, *L*_2,δ_  =13.39 months | | | VE_0_=63.1%, *L*_2,ο_ =2.86 months | | |  |
| Variant | Alpha |  | Delta | | | Omicron | | | Gram et al 2022 [20] |
| Two doses (BNT162b2 mRNA or mRNA-1273) | | | | | | | | |  |
| Initial VE | 91% |  | 87.2% | | | 40.0% | | |  |
| Mean duration | 16.39 months |  | 10.64 months | | | 4.02 months | | |  |
| **3 doses** |  |  |  |  |  |  |  |  | Lau et al 2023 [26] |
| Vaccine |  |  |  |  |  | BNT162b2 | CoronaVac |  |  |
| Initial VE |  |  |  |  |  | 48% | 30% |  |  |
| Mean duration |  |  |  |  |  | 5.08 months | 1.92 months |  |  |
| Average |  |  |  |  |  | Initial VE=39%, *L*_2,ο_ =2.78 months | | |  |
| Overall-one dose |  | | VE_0_= 53.9%, *L*_1,δ_=10.20 months | | | VE_0_= 36.1%, *L*_1,ο_ =1.40 months | | |  |
| Overall—full vaccination | VE_0_=(0.97+0.91)/2=0.94*L*_2,α_ = (6.71+16.39)/2 = 11.6 months, range (6.71,16.39) months | | VE_0_ = (0.765+0.865+0.827+0.93+0.894+0.872)/6 = 0.859; *L*_2,δ_ = (11.43+7.78+4.82+5.35+13.39+10.64)/6 =8.90 months, range (4.82,13.39) months | | | VE_0_ = (0.631+0.40+0.391)/3=0.473; *L*_2,ο_ = (2.86+4.02+2.78)/3=3.22 months, range (2.78,4.02) months | | |  |

**Technical details of Model and Methods**

The model we proposed to describe the COVID-19 epidemic outbreaks in Laos included two components: transmission dynamics with vaccination and replacement of SARS-CoV-2 variants, and disease reporting as depicted in Fig 2**.**

**Transmission model**

During the transmission process, a susceptible person (*S*) contracts the infection from infectious persons under the force of infection λ and then enters the latent class (*E*); after a latent period of *L*, the exposed person progresses to become infectious (*I*) for a period of *D* and can transmit the SAR-COV-2 virus, before recovering (*R*) or dying. After an immunity duration of *L*_im0_, the immune person wanes to enter a compartment *W*. To reflect that the distributions of both latent period (*L*) and infectious period (*D*) are of gamma distribution rather exponential distribution [27,28], the latent period and infectious period are divided into two equal parts, respectively, representing the numbers of people in two latent periods as *E*_1_(*t*) and *E*_2_(*t*), and the numbers of people in two infectious periods as *I*_1_(*t*) and *I*_2_(*t*). People who have been vaccinated (*V*_1_ singly or *V*_2_ fully) acquire partial protection against infection and can still be infected at a reduced rate of infection. $E_{1}^{V1}$, $E_{2}^{V1},$and $I_{1}^{V1}$, $I_{2}^{V1}$ are the corresponding infected states for those vaccinated once; $E_{1}^{V2}$, $E_{2}^{V2},$and, $I_{1}^{V2}$, $I_{2}^{V2}$ are the corresponding infected states for those fully vaccinated. We assumed that people randomly mix by ignoring the effects of population structure and geographical heterogeneity. The transmission dynamics was approximated by equation (S1),

$\frac{d}{dt}S\left( t \right)=-$λ$\left( t \right)S\left( t \right)-V_{1}(t)(t-t{}_{n})$

$$\frac{d}{dt}V_{1}\left( t \right)=-\left( t \right)\left( 1-{VE}_{1}(t) \right)V_{1}\left( t \right)-\frac{V_{1}\left( t \right)}{L_{im,1}(t)}+V_{1}\left( t \right)\left( t-t{}_{n} \right)-V_{2}(t)(t-t{}_{n})$$

$$\frac{d}{dt}V_{2}\left( t \right)=-\left( t \right)\left( 1-{VE}_{2}(t) \right)V_{2}\left( t \right)-\frac{V_{2}\left( t \right)}{L_{im,2}(t)}+V_{2}\left( t \right)\left( t-t{}_{n} \right)$$

$$\frac{d}{dt}E_{1}\left( t \right)=\left( t \right)S\left( t \right)-E_{1}\left( t \right)$$

$\frac{d}{dt}E_{2}\left( t \right)=(E_{1}\left( t \right)-E_{2}(t))$

$$\frac{d}{dt}I_{1}\left( t \right)=imported\left( t \right)+E_{2}\left( t \right)-I_{1}\left( t \right)$$

$$\frac{d}{dt}I_{2}\left( t \right)=(I_{1}\left( t \right)-I_{2}\left( t \right))$$

$$\frac{d}{dt}E_{1}^{V1}\left( t \right)=\left( t \right){\left( 1-{VE}_{1}\left( t \right) \right)V}_{1}(t)-E_{1}^{V1}\left( t \right)$$

$\frac{d}{dt}E_{2}^{V1}\left( t \right)=(E_{1}^{V1}\left( t \right)-E_{2}^{V1}(t))$ (S1)

$$\frac{d}{dt}I_{1}^{V1}\left( t \right)=E_{2}^{V1}-I_{1}^{V1}$$

$$\frac{d}{dt}I_{2}^{V1}\left( t \right)=(I_{1}^{V1}\left( t \right)-I_{2}^{V1}\left( t \right))$$

$$\frac{d}{dt}E_{1}^{V2}\left( t \right)=\left( t \right){[\left( 1-{VE}_{2}\left( t \right) \right)V}_{2}(t)+(t)W(t)]-E_{1}^{V2}\left( t \right)$$

$\frac{d}{dt}E_{2}^{V2}\left( t \right)=(E_{1}^{V2}\left( t \right)-E_{2}^{V2}(t))$

$$\frac{d}{dt}I_{1}^{V2}\left( t \right)=E_{2}^{V2}-I_{1}^{V2}$$

$$\frac{d}{dt}I_{2}^{V2}\left( t \right)=(I_{1}^{V2}\left( t \right)-I_{2}^{V2}\left( t \right))$$

$$\frac{d}{dt}R\left( t \right)={(I}_{2}\left( t \right)+I_{2}^{V1}+I_{2}^{V2})-\frac{R\left( t \right)}{L_{im,0}(t)}$$

$$\frac{d}{dt}W\left( t \right)=\frac{R\left( t \right)}{L_{im,0}(t)}+\frac{V_{1}\left( t \right)}{L_{im,1}(t)}+\frac{V_{2}\left( t \right)}{L_{im,2}(t)}-(t)\left( t \right)W\left( t \right)$$

Here *N* (=7,389,060) is the population size of Laos in 2020 [29] and assumed to be constant during the outbreaks by ignoring births or deaths. *S*(*t*) is the number of susceptible people at time *t*. *V*_1_(t) and *V*_2_(t) represent the numbers of those who have only one dose of vaccine and have been fully vaccinated up to time *t*, respectively; their respective VE against SARS-CoV-2 infection is denoted by *VE*_1_ and *VE*_2_; ΔV_1_(*t_n_*) and ΔV_2_(*t_n_*) represent their increases on day *t_n_* =1, 2.,3…. δ(*t*-*t*_n_) is Kronecker delta function: δ(*t*-*t*_n_) = 1 if *t* = *t*_n_, and =0 otherwise. It is assumed that the recovered (*R*) and the vaccinated (*V*_1_ and *V*_2_) lose their immunity at rates 1/*L*_im,0_, 1/*L*_im,1_ and 1/*L*_im,2_, respectively. *W*(*t*) represents the number of those who lost immunity acquired through previous infections or vaccination and therefore can be re-infected or breakthrough infected; their relative susceptibility is denoted by parameter *σ*. The rationale to put those naturally infected or vaccinated into one compartment *W* is that VE of two doses remains at least as great as protection afforded by prior natural infection [12]. For simplicity, the vaccinated with 1 or 2 doses are assumed to be approximated similarly. In equation (S1), *κ*=2*/L* and *γ*=2/*D*.

We considered the effect of vaccines on transmission of SARA-CoV-2 variants [30]. Compared to the unvaccinated individuals, infectiousness of the vaccinated individuals during subsequent infection episodes was assumed to reduce so the force of infection in equation (S1) was given as

$\lambda(t)=\frac{\beta\left( t \right)}{N}\left[ I_{1}\left( t \right)+I_{2}\left( t \right)+\varepsilon_{1}\left( t \right)\left( I_{1}^{V1}\left( t \right)+I_{2}^{V1}\left( t \right) \right)+\varepsilon_{2}\left( t \right)\left( I_{1}^{V2}\left( t \right)+I_{2}^{V2}\left( t \right) \right) \right]$ (S2)

Here *ε*_1_(*t*) and *ε*_2_(*t*) were the respective relative infectiousness of those having one dose of vaccine and those having fully vaccinated (see equation (S6) below).

As importation of COVID-19 cases continued along the epidemic outbreaks in Laos (Fig 1a), an item *imported*(t) was included in equation (S1) for the group of infectious people (*I*_1_). Separating imported cases from the local cases can help accurately estimate the transmissibility of SARS-CoV-2 [31,32,33].

During the period from 11 April 2021 to 12 May 2022, three variants of concern: Alpha, Delta and Omicron have been circulated and replaced one by another consequently. Delta variant first emerged from importation to Laos from 7^th^ of July 2021 (day τ_α_= 86 from 11 April 2021) and Omicron variant first reported from importation from 1^st^ of February 2022 (day τ_δ_= 296 from 11 April 2021) (WHO website [34], Fig 1c). To reflect the overtake processes of one variant by another variant, it was simply assumed that VE against SARS-CoV-2 infection changed in a sigmoidal fashion [35],

${VE}_{i}\left( t \right)=e_{i,\alpha}$ when 1≤t<τ_α_

${VE}_{i}\left( t \right)=e_{i,\alpha}+\frac{e_{i,\delta}-e_{i,\alpha}}{1+\exp\left( -\left( t-\tau_{*} \right)C_{\delta} \right)}$ when τ_α_≤t<τ_δ_ (S3)

${VE}_{i}\left( t \right)=e_{i,\delta}+\frac{e_{i,o}-e_{i,\delta}}{1+\exp\left( -\left( t-\tau_{*} \right)C_{o} \right)}$ when τ_δ_≤t

The three parameters *e_i_*_,α_, *e_i_*_,δ_ and *e_i_*_,ο_ represented VE against infection of Alpha, Delta, and Omicron variants, respectively, with subscript *i* representing number of doses of vaccine: *i*=1 for one dose of vaccine, *i*=2 for fully vaccinated. *τ*_δ*,_ *τ*_ο*_ were the midpoint of transition from Alpha variant to Delta variant, and from Delta variant to Omicron variant, respectively. *C*_δ_ and *C*_ο_ were the rate parameters that controlled the speed of the corresponding transitions. With a speed parameter *C*, the sigmoidal function can more flexibly and better fit the actual change than the step function. In this study, we fixed VE against Alpha and Delta variants at *e*_1,α_=72%, *e*_2,α_=86% [10], *e*_1,δ_ =50.5% and *e*_2,δ_ =74.5% [12] but let *e_i_*_,ο_ to be inferred from model calibration to data. In view of variation in the estimates of VE among different vaccines and different populations (Tables S4 and S5), the sensitivity analysis was conducted on different fixed values of *e*_1,α_, *e*_2,α_, *e*_1,δ_ and *e*_2,δ_ such as that increasing 10% and decreasing 15% in absolute values: 82%, 96%, 60.5%, 84.5%; or 57%,71%, 35.5%, 59.5% for the parameters (see **Sensitivity analyses**).

In the same way, the waning rate of protection (i.e., the inverse of the duration of protection) was assumed to change in a sigmoidal fashion as

$\frac{1}{L_{im,i}(t)}=\frac{1}{L_{i,}(t)}$ when 1≤t<τ_α_

$\frac{1}{L_{im,i}(t)}=\frac{1}{L_{i,}(t)}+\frac{\frac{1}{L_{i,}(t)}-\frac{1}{L_{i,}(t)}}{1+\exp\left( -\left( t-\tau_{*} \right)C_{\delta} \right)}$ when τ_α_≤t<τ_δ_ (S4)

$\frac{1}{L_{im,i}(t)}=\frac{1}{L_{i,}(t)}+\frac{\frac{1}{L_{i,}(t)}-\frac{1}{L_{i,}(t)}}{1+\exp\left( -\left( t-\tau_{*} \right)C_{o} \right)}$ when τ_δ_≤t

Following the empirical studies (Table S5), we assumed as a baseline that *L*_i,α_ =354 days, *L*_i, δ_ =273 days, *L*_i,ο_ =98 days for *i*=0 (natural infection), and 2 (fully vaccinated). For simplicity we assumed that the duration of immunity acquired from single vaccination was half of that from full vaccination: *L*_1,α_ = *L*_2,α_/2=177 days, *L*_1, δ_ = *L*_2, δ_ /2 =136 days, *L*_1,ο_ = *L*_2,o_ /2=49 days. Sensitivity analysis was conducted for other choices of the parameter values (see **Sensitivity analyses**). We further fixed the latent period (=incubation period) at *L*=5.2 days [36,37,38] and infectious period at *D* =3.5 days [39].

Similarly, the risk of re-infection of the people in compartment *W* (i.e., susceptibility of *W* to infection of Alpha, Delta, and Omicron variants) was assumed to change as

$\left( t \right)=0.16 (=_{\alpha})$ when 1≤t<τ_α_

$\left( t \right)={}_{\alpha}+\frac{{}_{\delta}-{}_{\alpha}}{1+\exp\left( -\left( t-\tau_{*} \right)C_{\delta} \right)}$ when τ_α_≤t<τ_δ_ (S5)

$\left( t \right)={}_{\delta}+\frac{{}_{o}-{}_{\delta}}{1+\exp\left( -\left( t-\tau_{*} \right)C_{o} \right)}$ when τ_δ_≤t

The value of *σ*_α_ was fixed at 0.16 [40,41], and the values of *σ*_δ_ and *σ*_ο_ were estimated from model calibration.

The infectiousness of vaccinated individuals during subsequent infection episodes relative to the unvaccinated was assumed to vary along the course of outbreak as

${}_{i}\left( t \right)={}_{i,\alpha}$ when 1≤t<τ_α_

${}_{i}\left( t \right)={}_{i,\alpha}+\frac{{}_{i,\delta}-{}_{i,\alpha}}{1+\exp\left( -\left( t-\tau_{*} \right)C_{\delta} \right)}$ when τ_α_≤t<τ_δ_ (S6)

${}_{i}\left( t \right)={}_{i,\delta}+\frac{{}_{i,o}-{}_{i,\delta}}{1+\exp\left( -\left( t-\tau_{*} \right)C_{o} \right)}$ when τ_δ_≤t

According to [30], we fixed relative infectiousness of those having fully vaccinated at *ε*_2,α_ = 40% and *ε*_2,δ_ =63% for Alpha and Delta variants, respectively. Note that the values cited here are the average over the two vaccines surveyed in England: BNT162b2 and ChAdOx1. The relative infectiousness of those having fully vaccinated *ε*_2,ο_ for Omicron variant was inferred from model calibration. For simplicity, we assumed that relative infectiousness of those having one dose of vaccine was equal to that of those having fully vaccinated (i.e., *ε*_1,._= *ε*_2,._) but other potential relationships between *ε*_1,._and *ε*_2,._ such as *ε*_1,._= (1+0.3)*ε*_2,._ was assessed in the sensitivity analysis (See **Sensitivity analyses**)

Case fatality rate (CFR), which was defined as the proportion of mortality among the infections confirmed and reported, was also assumed to change with the consecutive replacement of Alpha, Delta, and Omicron variants in the Laos population as

$CFR\left( t \right)=CFR_{\alpha}$ when 1≤t<τ_α_

$CFR\left( t \right)=CFR_{\alpha}+\frac{CFR-CFR_{\alpha}}{1+\exp\left( -\left( t-\tau_{*} \right)C_{\delta} \right)}$ when τ_α_≤t<τ_δ_ (S7)

$CFR\left( t \right)=CFR+\frac{CFR-CFR}{1+\exp\left( -\left( t-\tau_{*} \right)C_{o} \right)}$ when τ_δ_≤t

In view of the varying control policies and capabilities of Laos’ health authorities over the outbreaks, we considered two assumptions on ascertainment rate (ASC): the first was that there were two turning points *τ*_asc1_ and *τ*_asc2_ such that

$ASC\left( t \right)=ASC_{1}$ when t<τ_asc1_

$ASC\left( t \right)=ACS_{2}$ when τ_asc2_≥t>τ_asc1_ (S8a)

$ASC\left( t \right)=ACS_{3}$ when t≥τ_asc2_

And the second one was that there were three turning points *τ*_asc1_, *τ*_asc2_ and *τ*_asc3_ such that

$ASC\left( t \right)=ASC_{1}$ when t<τ_asc1_

$ASC\left( t \right)=ACS_{2}$ when τ_asc2_≥t>τ_asc1_ (S8b)

$ASC\left( t \right)=ACS_{3}$ when τ_asc3_≥t>τ_asc2_

$ASC\left( t \right)=ACS_{4}$ when t≥τ_asc3_

By calibrating the models under these two assumptions to observational data, the better assumption assessed by deviance information criterion (DIC) was selected (see **Assessment of assumptions about ascertainment and contact mechanisms**).

During the period from 11 April 2021 to 12 May 2022 when variant replacing one by another among Alpha, Delta, and Omicron, the transmission coefficient (*Β*) of infection was assumed to change as

$\left( t \right)={}_{\alpha}$ when 1≤t<τ_α_

$\left( t \right)={}_{\alpha}+\frac{{}_{\delta}-{}_{\alpha}}{1+\exp\left( -\left( t-\tau_{*} \right)C_{\delta} \right)}$ when τ_α_≤t<τ_δ_ (S9)

$B\left( t \right)=B_{\delta}+\frac{B_{o}-B_{\delta}}{1+\exp\left( -\left( t-\tau_{*} \right)C_{o} \right)}$ when τ_δ_≤t

Combining temporal variations in transmission coefficient *Β* of infection and contact rates recorded and reflected by Google mobility data [42] or incurred by Government control policy, the effective contact rate was assumed to be approximated as

$\beta\left( t \right)=B\left( t \right)(GM\_WeeklyAverage(t)+100)/100$ (S10a)

$\beta\left( t \right)=B\left( t \right)(100-GOVIndex(t))/100$ (S10b)

Here GM_WeeklyAverage was the seven-day average of the overall average of visits at six locations: retail_and_recreation, grocery_and_pharmacy, parks, transit_stations, workplaces and residential (Fig 1a; [1]). GOVIndex, government response index, was a composite measure provided by the Oxford COVID-19 Government Response Tracker (OxCGRT) (Fig 1; [2]) based on 16 indicators, including eight containment and closure policy indicators (school closures, workplace closures, public event cancellations, gathering restrictions, public transport closures, stay-at-home orders, internal movement restrictions, and international travel controls), two economic response (income support and debt/contract relief for households), six health systems indicators (public information campaign, testing policy, contact tracing, facial coverings, vaccination policy and protection of elderly people), scaled range from 0 (no interventions) to 100 (implementing the strictest NPIs). [Sarracino](https://sites.google.com/site/fsarracino/) et al [43] showed that the association between the Response Index and mobility (time at home) over time was mostly positive. By calibrating the models under these two contact mechanisms to observational data, the better contact mechanism assessed by DIC was selected (see **Assessment of assumptions about ascertainment and contact mechanisms**).

To monitor the change of epidemic features during the period under investigation, we calculated the susceptibility as

Susceptibility(*t*) = (*S*(*t*)+(1-*VE*_1_(*t*))*V*_1_(*t*-ξ)+(1-*VE*_2_(t))*V*_2_(*t*-ξ)+*σ*(t)*W*(*t*))/*N* (S11)

and the time-varying reproduction number as

*R*_t_(*t*) = Susceptibility(*t*)×β(*t*)×D (S12)

In equation (S11), it was assumed that protection acquired from vaccination took effect after ξ days of injection. In view of vaccination survey study [44,45,46], we assumed ξ=14 days.

**Initial seeding:** We considered the epidemic starting from 11^th^ April 2021 on which there were *I*_0_ infections. The initial condition was parametrized as follows (c.f.,[28]),

$$I_{1}\left( 0 \right)=\frac{I_{0}}{1+{}/{(+\Psi_{r})}}$$

$$I_{2}\left( 0 \right)=I_{0}-I_{1}(0)$$

$E_{2}(0)=I_{1}(0){(\Psi}_{r}+)$

$E_{1}\left( 0 \right)=E_{2}(0){(\Psi}_{r}+)$ (S13)

$$V_{1}\left( 0 \right)=V_{1}^{*}$$

$$V_{2}\left( 0 \right)=V_{2}^{*}$$

$$S\left( 0 \right)=N-I_{1}\left( 0 \right)-I_{2}\left( 0 \right)-E_{1}\left( 0 \right)-E_{2}\left( 0 \right)-V_{1}^{*}-V_{2}^{*}$$

and all other compartments were initially empty. Here $V_{1}^{*}$ and $V_{2}^{*}$ represent the cumulative numbers of people having one dose of vaccine and people having fully vaccinated on 29 March 2021 (two weeks earlier) (Fig S1b), respectively, and *ψ*_r_ represented the initial growth rate, which determined the first transmission coefficient as

${}=\Psi_{r}\frac{{(L{\Psi_{r}}/2+1)}^{2}}{1-{(D{\Psi_{r}}/2+1)}^{-2}}$ (S14)

**Disease reporting**

For disease reporting process, we assumed that only a proportion *ASC* (ascertainment rate) of all infected people were identified and reported. The confirmed infections either died (with probability *CFR—case fatality rate*) or recovered. In this study we simply assumed that the incubation was equal to the latent period. For the infections that were identified, the delay from symptom onset to confirmation was described by a gamma distribution *f*_Confirm|INF_ with mean = m_OC_ days and variance = v_OC_ days^2^. The mean number of daily new confirmations was produced as

$\boldsymbol{\mu}_{\mathbf{Confirm}}\boldsymbol{(t)=ASC(t)}\sum_{\boldsymbol{s=0}}^{\boldsymbol{t}} \boldsymbol{f}_{\mathbf{Confirm|INF}}\boldsymbol{(t-s)\times\Delta I(s)}$ (S15)

Here $\Delta I\left( t \right)$represented the daily number of new infectious cases on day *t* generated from equation (S1) as,

$\Delta I\left( t \right)=\int_{t-1}^{t} \frac{2}{L}{(E}_{2}\left( s \right)+E_{2}^{V1}(s)+E_{2}^{V2}(s))ds$ (S16)

The delay from confirmation to death was approximated by a gamma distribution $f_{\mathrm{Death}|\mathrm{Confirm}}$ with mean = m_CD_ days and variance = v_CD_ days^2^. The mean daily number of new deaths was generated as

$\boldsymbol{\mu}_{\mathbf{Death}}\boldsymbol{(t)=CFR(t)}\sum_{\boldsymbol{s=0}}^{\boldsymbol{t}} \boldsymbol{f}_{\mathbf{Death|Confirm}}\boldsymbol{(t-s)\times}{}_{\mathrm{Confirm}}\boldsymbol{(s)}$ (S17)

The recovered patients were

$\boldsymbol{\mu}_{\mathbf{Recovery}}\boldsymbol{(t)=(1-CFR(t))}\sum_{\boldsymbol{s=0}}^{\boldsymbol{t}} \boldsymbol{f}_{\mathbf{Recovery|Confirm}}\boldsymbol{(t-s)\times}{}_{\mathrm{Confirm}}\boldsymbol{(s)}$ (S18)

Here the delay from confirmation to recovery was approximated by a gamma distribution $f_{\mathrm{Recover}|\mathrm{Confirm}}$ with mean = m_CR_ days with variance = v_CR_ days^2^.

In view of the available estimates from China data [47], we simply fixed the six parameters at m_OC_=4.75 days, v_OC_=4.7 days^2^, m_CD_=19.5 days, v_CD_=22.7 days^2^, m_CR_=15.4 days, v_CR_=15.5 days^2^. Once the six parameters were given, all delay distributions were discretised into densities among 45 days as pgamma(*i*;shape,rate)-pgamma(*i*-1;shape,rate), *i*=,1,2,…,45. Here pgamma is the incomplete gamma function defined in R [48].

**Inference model**

In the inference of model parameters, 7-day central averages of the originally observed dataset of confirmed cases and deaths were used to avoid and reduce the effects of potential delays and weekly patterns of reporting. The negative binomial likelihood function was assumed to accommodate variation in the daily number of new cases. The observed number *x*^C^ (*t*) of cases on day *t* was assumed to distribute as

$l(x^{\text{C}}(t)|\Theta,\eta)=\frac{\Gamma(x^{\text{C}}(t)+r^{\text{C}}(t))}{\Gamma(r^{\text{C}}(t))\Gamma(x^{\text{C}}(t)+1)}(\frac{1}{\eta})^{r^{\text{C}}(t)}(1-\frac{1}{\eta})^{x^{\text{C}}(t)}$ (S19a)

where

$r^{C}\left( t \right)=\frac{\mu(t)}{\eta-1}$ (S19b)

Here $\eta$ was the dispersion parameter and $\mu(t)$ were the model predictions of the cases on day *t* (i.e., equations (S15) and (S17)). We assumed that variations in daily numbers of confirmed infections and deaths followed negative binomial distributions with different dispersion parameters: *η*_Case_ and *η*_Death_, respectively. As recovery data was only available until August 4, 2021, the cumulative number of recoveries on 4 August 2021 was assumed to follow Poisson distribution:

$l\left( Recovery | \Theta\right)=\frac{\exp(-Z)}{\mathrm{Recovery}!}Z^{Recovery}$ (S19c)

Here *Recovery* represented the cumulative number of the recovered reported on August 4, 2021 (day 115 from 11 April 2021), and $Z=\int_{1}^{115} \mu_{Recovery}(s)ds$ represented the mean from equation (S18).

Twenty five parameters to be estimated were: **Θ** ={*I*_0_, ψ_r_,*τ*_α_^*^, *B*_δ_, *τ*_δ_^*^, *B*_ο_, *σ*_δ_, *σ*_ο_, *ε*_2,ο_, CFR_α_, CFR_δ_, CFR_ο_, *e*_1,ο_, *e*_2,ο_, *C*_δ_, *C*_ο_, ASC_1_, τ_ASC1_,ASC_2_,τ_ASC2_, ASC_3_,τ_ASC2_, ASC_4_,*η*_Case_, *η*_Death_}(or 23 parameters if assuming two turning points in ASC). Assuming that the observed daily number of confirmed cases: *x*(1), *x*(2),…, *x*(*T*), and daily number of deaths: *y*(1), *y*(2), …,*y*(*T*) were conditionally independent, the total likelihood given model parameters **Θ** was

$L\left( \Theta,\eta_{Case},\eta_{Death} \right)=l\left( Recovery | \Theta\right)\prod_{t=1}^{T} l\left( x\left( t \right) | \Theta,\eta_{Case} \right)l\left( y\left( t \right) | \Theta,\eta_{Death} \right)$ (S20)

*Parameter inference*: We assumed the uninformative prior distributions *f*(**Θ**) which were uniform for parameters (Table 1). Employing Bayesian framework through the combination of the prior distribution *f*(**Θ**) and the likelihood *L*(**Θ**,*η_Case_,η_Death_*), the posterior distribution can be obtained by Markov Chain Monte Carlo simulations (MCMC) [33]. A normal random walk was used to sample the new proposal for each element of **Θ**; the proposal was accepted or rejected as the next step of the Markov chain with the probability regulated by the Bayesian law. To generate nearly independent samples of model parameters, the samples were to be thinned every 300^th^ observations. To respond to the acceptance rate, the following adaptive procedure was applied: if the acceptance ratio over 300 × 50 iterations was less than 12%, then decrease the jump step to 80% of its current size; if it exceeded 40%, then the jump step increased to 120% of its current size. Otherwise, the jump step remained unchanged. To allow the MCMC process to fully converge, a burn-in period of 800,000 iterations was chosen, and the estimates of model parameters were obtained from the further 1,500,000 iterations. From these samples, we can obtain medians and their 95% credible intervals for model parameters.

*Model comparison and selection*: Equations (S8) and (S10) allowed for model variants that differed in contact mechanisms and temporal change in ASC. To select the best model variant, DIC, which combines the goodness of fit and model complexity [49], was used to compare the performance of those model variants. It measures fit via the deviance Dev(Θ) = −2log*L*(Θ|Data) and complexity by an estimate of the ‘effective number of parameters’ *p*_D_ = mean(Dev(Θ)) − Dev(mean(Θ)) (i.e., posterior mean deviance minus deviance evaluated at the posterior mean of the parameters). The DIC is calculated as:

DIC = Dev(mean(Θ)) + 2*p*_D_ = mean(Dev(Θ)) + *p*_D_. (S21)

The model that has the smallest DIC is the best.

**Assessment of assumptions about ascertainment and contact mechanisms**

We compared the performance of our model under three different conditions: 1) temporal change in contact rate driven by Google mobility and ASC changed at three turning points; 2) temporal change in contact rate driven by Google mobility and ASC changed at two turning points; and 3) temporal change in contact rate driven by Government response index and ASC changed at three turning points. The model under the three situations could generate the observational outbreak data of confirmed case and deaths (Fig S3). Under the two temporal change patterns of ASC, the estimates of transmissibility and CFR of the three variants of SARS-CoV-2 virus were comparable but the estimates of ASC were different. By the values of DIC (5097 for 3 turning points in ASC versus 5291 for 2 turning points in ASC; Table S6), the model assuming a temporal pattern of ASC with three turning points performed much better. For the two contact mechanisms, their estimates of CFR and other features were comparable, but the estimates of transmission coefficients and ASCs were quite different. The differences should be a result of the distinct mechanisms assumed for the contact rate. The values of DIC (5097 for Google mobility contact versus 5445 for Government index contact) indicated that the model assuming contact rate driven by Google mobility was much better. The comparison indicated that the Laos population compliance with government policies was not high and stable (c.f.,[50]) but Google mobility data could reliably reflect the behavioral changes. These tests provided support for the use of our model with contact rate driven by Google mobility and ASC changed at three turning points during the outbreak period.

The data described and codes compiled for modelling analyses in this article can be freely and openly accessed at Laos COVID-19 modelling: [https://github.com/wlx0871/laosCOVID19](https://github.com/wlx0871/laosmodellingCOVID19)modelling [51].


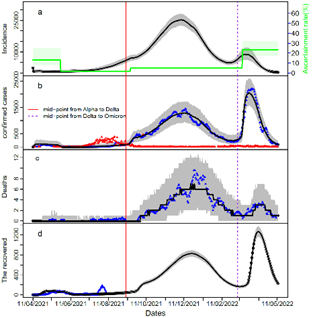

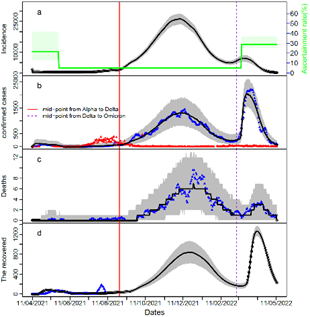

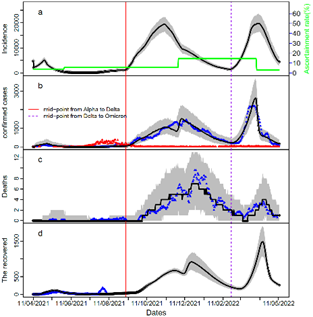


Fig S3 Model estimation of infections and model fit to daily number of local cases, deaths, and recoveries during COVID-19 outbreaks in Laos from 11 April 2021 to 12 May 2022. Left panel) contact rate controlled by Google mobility with 3 turning points in ASC; middle panel) contact rate controlled by Google mobility with 2 turning points in ASC; right panel) contact rate controlled by Government response index with 3 turning points in ASC. (a) the daily number of new local infections, (b) the daily number of new local confirmed cases, (c) daily number of new deaths, and (d) daily number of new recovered. The thick black line represented median predictions and grey shading is their 95% credible intervals, with the blue triangles representing the 7-day averages of observational data. The green line in panel (a) represented the ASC. The red dots in panel (b) represented the daily numbers of imported cases. The red and purple vertical lines stood for the midpoints of transition from Alpha to Delta variant and from Delta to Omicron variant, respectively. The recovery data in panel (d) were available up to day 116 (i.e., 4 August 2021).

**Table S6:** Model comparison and selection among contact mechanisms and ascertainment assumptions. The following model parameters were fixed: relative infectivity for Alpha variant of the fully vaccinated (ε_2,α_) =40%, relative infectivity for Delta variant of the fully vaccinated (ε_2,δ_) =63% [30] and ε_1,α_=ε_2,α,_ ε_1,δ_=ε_2,δ_ were assumed; for VE, e_1,α_=72%, e_2,α_ =86% [10]; e_1,δ_ = 50.5%, e_2, δ_  =74.5% [12].

| **Parameter** | **Prior** | **Posterior (median[95%CrI])** | | |
| --- | --- | --- | --- | --- |
|  |  | Contact change via Google Mobility Eq (S10a) | | Contact change via response index Eq (S10b) |
|  |  | 4ASC (eq(S8b)) | 3ASC (Eq(S8a)) | 4ASC (Eq(S8b)) |
| Initial number of seeds (I_0_) | U[100,6000] | 2404[1170,4354] | 1671[794,2819] | 4896[4524,4995] |
| Initial growth rate (ψ_r_) | U[0.02,0.40] | 0.059[0.052,0.068] | 0.050[0.045,0.058] | 0.15[0.15,0.160] |
| Transmission coefficient of Alpha variant (B_α_) | – | 0.44[0.42, 0.47] | 0.41[0.40,0.44] | 0.80[0.77,0.82] |
| Basic reproduction number of Alpha variant (R_0,α_) | – | 1.55[1.47,1.64] | 1.45[1.40,1.53] | 2.79[2.70,2.88] |
| Transmission coefficient of Delta variant (B_δ_) | U[0.50,5.00] | 0.83[0.81,0.87] | 0.79[0.78,0.80] | 1.96[1.92,1.99] |
| Transmission coeff. of Omicron (B_ο_) | U[0.80,15.0] | 2.77[2.36,3.13] | 2.27[1.96,2.54] | 9.34[8.53,10.15] |
| midpoint in transition from Alpha to Delta variant (τ_α*_) | U[5 Jul, 13 Oct 2021] | 09 [06,16] Sep 2021 | 31 Aug[30 Aug, 3 Sep] 2021 | 7 [6,7] Sep 2021 |
| midpoint in transition from Delta to Omicron variant (τ_δ*_) | U[5 Feb, 1 Apr 2022] | 5 Mar [20 Feb, 11 Mar 2022] | 6 Mar[25 Feb, 13 Mar] 2022 | 24 [22,25] Feb 2022 |
| Susceptibility of W to Delta variant (σ_δ_) | U[0.13,0.70] | 0.64[0.47, 0.697] | 0.67[0.57,0.699] | 0.69[0.67,0.70] |
| Susceptibility of W to Omicron variant (σ_ο_) | U[0.13,0.60] | 0.136 [0.13,0.165] | 0.143[0.131,0.191] | 0.134[0.13,0.148] |
| Relative infectivity for Omicron of the singly vaccinated (ε_1ο_) | ε_1ο_=ε_2ο_ | 0.26[0.25,0.28] | 0.26[0.25,0.29] | 0.26[0.25,0.28] |
| Relative infectivity for Omicron of the fully vaccinated (ε_2ο_) | U[0.25,1.00] | 0.26[0.25,0.28] | 0.26[0.25,0.29] | 0.26[0.25,0.28] |
| ASC_1_ (%) | U[1,95] | 13.20[7.98,25.04] | 21.56[12.81,41.73] | 3.93[3.40,4.55] |
| First turning point in ASC (τ_ASC1_) | U[10 May,19 Jun 2021] | 21 May[18, 25 May 2021] | 22 May[17, 28] May 2021 | 25 May[10 May, 13 Jun] 2021 |
| ASC_2_ (%) | U[1,95] | 1.81[1.26,2.40] | 5.02[4.76,5.39] | 5.54[5.03,6.23] |
| Second turning point in ASC (τ_ASC2_) | U[20 Jun,15 Dec 2021] | 14 [11, 17] Sep 2021 | 14 [13,16] Mar 2022 | 29 Nov [25 Nov, 2 Dec] 2021 |
| ASC_3_ (%) | U[1,95] | 5.15[4.80,5.66] | 28.72 [25.60,36.72] | 14.48 [13.04,16.51] |
| Third turning point in ASC (τ_ASC3_) | U[1 Jan,5 Apr 2022] | 14 [13,16] Mar 2022 | – | 5 [4,5] Apr 2022 |
| ASC_4_ (%) | U[1,95] | 23.44[17.17,31.98] | – | 3.06[2.42,3.74] |
| CFR of Alpha variant (%) ^♦^ | U[cfr_0_/5,5cfr_0_] | 0.38[0.21, 0.60] | 0.45[0.27,0.71] | 0.52[0.33,0.75] |
| CFR of Delta variant (%) ^♦^ | U[cfr_0_/5,5cfr_0_] | 1.05[0.96,1.15] | 1.03[0.94,1.12] | 1.02[0.93,1.11] |
| CFR of Omicron variant (%) ^♦^ | U[cfr_0_/5,5cfr_0_] | 0.28[0.18,0.39] | 0.25[0.14,0.35] | 0.37[0.30,0.45] |
| Dispersion parameter for cases (η_Case_) | U[5,200] | 32.14 [27.71,37.22] | 35.83 [30.66,41.68] | 43.66 [37.35,50.59] |
| Dispersion parameter for deaths (η_Death_) | U[1.01,5.00] | 1.008 [1.005,1.021] | 1.009 [1.005,1.026] | 1.013 [1.01,1.025] |
| VE of 1 dose against Omicron variant e_1,ο_ (%) | U[5,50] | 44.93 [24.32,49.80] | 42.56 [13.51,49.73] | 42.42 [14.84,49.78] |
| VE of full doses against Omicron variant e_2,ο_ (%) | U[10,75] | 72.72 [62.26,74.90] | 71.52 [56.86,74.86] | 73.83 [69.94,74.95] |
| Replacement rate of Alpha by Delta variant (C_δ_) | U[0.05,2.0] | 0.071 [0.055,0.104] | 1.20 [0.226,1.963] | 1.555 [0.768,1.979] |
| Replacement rate of Delta by Omicron variant (C_ο_) | U[0.05,2.0] | 0.127 [0.089,1.636] | 0.106 [0.073,0.178] | 1.429 [0.576,1.984] |
| DIC |  | 5097.43 | 5291.53 | 5445.13 |

^♦^cfr_0_ = 0.44% was the naïve estimate by dividing the total number of deaths by the total of cases reported. CFR was defined as the proportion of mortality among the infections confirmed and reported.

**Sensitivity analyses**

In the main text, the baseline scenario was defined as i) VE against Alpha and Delta variants fixed at *e*_1,α_=72%, *e*_2,α_=86%, *e*_1,δ_ =50.5% and *e*_2,δ_ =74.5% which were taken from the estimates of VE for BNT162b2 [12] and ChAdOx1 [10]; ii) the protection lasting *L*_i,α_ =354 days, *L*_i, δ_ =273 days, *L*_i,ο_ =98 days for *i*=0 (natural infection), and 2 (fully vaccinated), which were the median of the estimates from Table S5 and *L*_1,α_ =177 days, *L*_1, δ_ =136 days, *L*_1,ο_ = 49 days for the singly vaccinated; iii) infectivity of the singly vaccinated being equal to the infectivity of the full vaccinated, i.e., *ε*_1._= *ε*_2._. In this section we tested the robustness of our modelling results by investigating six different scenarios:

1. *Strong immunity* where VE against Alpha and Delta variants set at *e*_1,α_=82.0%, *e*_2,α_=96.0%, *e*_1,δ_ =60.5%, *e*_2,δ_ =84.5%, that is, VE increased by 10% in absolute value relative to the baseline scenario, and other conditions remained the same as in the baseline;

2. *Weak immunity* where VE against Alpha and Delta variants set at *e*_1,α_=57.0%, *e*_2,α_=71.0%, *e*_1,δ_ =35.5%, *e*_2,δ_ =59.5%, that is, VE decreased by 15% in absolute value, and other conditions remained the same as in the baseline;

3. *Stronger full vaccine effect on infectiousness* in which the singly vaccinated had relatively weak antibody and hence strong infectivity when getting infected afterwards set by *ε*_1._= min(1.0,1.3*ε*_2._) , and other conditions remained the same as in the baseline;

4. *Short immunity* where *L*_i,α_ =205 days, *L*_i, δ_ =147 days, *L*_i,ο_ =84 days for natural infection (*i*=0) and fully vaccination (*i*=2) and duration of immunity of single vaccination (*i*=1) being half of that of the full vaccination, that is, the lower bound of summary estimates from Table S5, and other conditions remained the same as in the baseline;

5. *Long immunity* where *L*_i,α_ =500 days, *L*_i, δ_ =408 days, *L*_i,ο_ =123 days for natural infection (*i*=0) and fully vaccination (*i*=2) and duration of immunity of single vaccination being half of that of the full vaccination, that is, the upper bound of summary estimates from Table S5, and other conditions remained the same as in the baseline;

6. *Equal duration of immunity* induced by single and full vaccination and natural infection *L*_i,α_ =354, *L*_i, δ_ =273 days, *L*_i, ο_ =98 days for *i*=0,1,2, and other conditions remained the same as in the baseline.

In view of the observations from Tables 1 and S6: the midpoint in transition from Alpha to Delta variant and the midpoint from Delta to Omicron variant were well converged at 9 Sep 2021 and 5 March 2022 (i.e., $\tau_{\alpha}^{*}$=152 and $\tau_{o}^{*}$=329), respectively; the relative infectiousness of these vaccinated was converged at 25% (ε_1,ο_=ε_2,ο_=0.25); susceptibility of the waned *W* to Delta and Omicron variants fixed at *σ*_δ_=70% and *σ*_δ_=13% (also see [40]). For quick convergence of the MCMC sampling, the model re-calibration was done with these six parameters fixed at these values under the above six sensitivity analysis scenarios, and the parameters to be inferred were

**Θ** ={*I*_0_, ψ_r_, *B*_δ_, *B*_ο_, CFR_α_, CFR_δ_, CFR_ο_, *e*_1,ο_, *e*_2,ο_, *C*_δ_, *C*_ο_, ASC_1_, τ_ASC1_, ASC_2_, τ_ASC2_, ASC_3_,τ_ASC2_, ASC_4_,*η*_Case_, *η*_Death_}

Our model was re-calibrated to the observational data under these different scenarios and the results were listed in Table S7 including the results for the baseline scenario under the new calibration for comparison. The estimates of model parameters for the baseline scenario under two calibrations (with 25 and 20 parameters to be inferred) remained nearly the same for the parameter to be inferred. It was shown that the three turning points in ASC consistently occurred around 21 May 2021, 14 September 2021 and 15 March 2022 under the six scenarios, respectively. Under *Weak immunity* against Alpha and Delta variants, *ASC*_4_ decreased from 19.9 % to 16.9% while the estimates of other parameters nearly remained the same as in the baseline: for example, transmission coefficient *B*_α_ of Alpha variant was nearly the same at 0.45 while *B*_δ_ decreased from 0.82 to 0.78 and *B*_ο_ increased from 3.00 to 3.33. Under *Strong immunity* against Alpha and Delta variants, *ASC*_1_ decreased from 15.4% to 11.1% and *ASC*_4_ increased from 19.9% to 23.5% while the other parameters remained nearly the same as in the baseline: for example, *B*_δ_ increased from 0.82 to 0.86 and *B*_ο_ decreased from 3.00 to 2.83. These indicated that the variation in VE against Alpha and Delta variants cannot significantly impact the epidemiological characteristics.

Under *Stronger full vaccine effect on infectiousness*, the estimates of the model parameters remained nearly the same as in the baseline with the relative changes being less than 5%. This may reflect the fact that among those vaccinated, most were fully vaccinated (4977532/5791016 =86%) and that the number of infections from these previously vaccinated was much less than those from unvaccinated.

Under *Short immunity* against Alpha and Delta variants, the parameter estimates did not much deviate from the baseline: for example, *B*_α_ slightly increased from 0.45 to 0.47, *B*_δ_ decreased from 0.82 to 0.79 and *B*_ο_ increased from 3.00 to 3.42, while *ASC*_1_ increased from 15.4% to 21.7% and *ASC*_3_ decreased from 5.0% to 4.2%. Under *Long immunity* against Alpha and Delta variants, much smaller deviation in parameter estimates from the baseline was observed with the largest deviation in *ASC*_1_ from 15.4% to 11.5% and *ASC*_4_ from 19.9% to 22.6%.

Under *Equal duration of immunity* against Alpha and Delta variants, all the estimates of model parameters were nearly the same as in the baseline with the largest relative change of 15.6% in ASC_1_.

Overall, the sensitivity analyses suggested the model calibrations remained stable under these different scenarios investigated. Further, the value of DIC implied that the best model scenario was the baseline with the smallest: DIC=5385.

**Table S7:** Parameters of the model incorporating Google mobility under different assumptions of duration of immunity. Here e_1,α_=0.72, e_2,α_=0.86, e_1,δ_=0.505, e_2,δ_ =0.745 and infectivity of the singly vaccinated (ε_1._) was assumed to be equal to that of the fully vaccinated (ε_2._), and for Alpha and Delta variant: ε_1,α_=ε_2,α_=0.4 and ε_1,δ_=ε_2,δ_=0.63, ε_1,ο_=ε_2,ο_=0.25. Further fixing midpoint in transition from Alpha to Delta variant (τ_α*_) on day 66+86=152 (9 Sep 2021) and midpoint in transition from Delta to Omicron variant (τ_δ*_) on day 296+33=329 (5 Mar 2022). Susceptibility of *W* to Delta (σ_δ_) =0.70; Susceptibility of *W* to Omicron (σ_ο_) =0.13.

| **Parameter** | **Prior** | **Posterior (median[95%CrI])** | | | | | | |
| --- | --- | --- | --- | --- | --- | --- | --- | --- |
|  |  | **Baseline** | Strong immunity | Weak immunity | Stronger full vaccine on infectiousness | Short immunity | Long immunity | Equal immunity for single and full vaccination |
| Initial number of seeds (I_0_) | U[100,6000] | 1997 [1306,3592] | 3029 [1410,5256] | 1838 [954,3294] | 2126 [1337,4542] | 1255 [806,2014] | 2872 [1779,4935] | 2418 [1174,5339] |
| Initial growth rate (ψ_r_) | U[0.02,0.4] | 0.061 [0.057,0.064] | 0.056 [0.053,0.062] | 0.063 [0.059,0.067] | 0.06 [0.056,0.064] | 0.070 [0.067,0.072] | 0.057 [0.053,0.060] | 0.060 [0.053,0.065] |
| Transmission coefficient of Alpha variant (B_α_) | – | 0.45 [0.43, 0.46] | 0.43 [0.42, 0.45] | 0.45 [0.44,0.47] | 0.44 [0.43,0.45] | 0.47 [0.46,0.48] | 0.43 [0.42,0.44] | 0.44 [0.42,0.46] |
| Initial basic reproduction number of Alpha variant (R_0,α_) | – | 1.56 [1.52,1.59] | 1.51 [1.47,1.57] | 1.59 [1.54,1.63] | 1.56 [1.51,1.59] | 1.65 [1.62,1.68] | 1.52 [1.48,1.55] | 1.55 [1.47,1.60] |
| Transmission coefficient of Delta variant (B_δ_) | U[0.50,5.00] | 0.82 [0.81,0.84] | 0.86 [0.83,0.88] | 0.78 [0.77, 0.81] | 0.82 [0.80,0.84] | 0.79 [0.78,0.80] | 0.84 [0.82,0.85] | 0.83 [0.80,0.87] |
| Transmission coefficient of Omicron variant (B_ο_) | U[0.8,15.0] | 3.00 [2.49,3.33] | 2.83 [2.34,3.20] | 3.33 [2.81,3.66] | 2.98 [2.64,3.27] | 3.42 [3.06,3.71] | 2.86 [2.49,3.17] | 2.94 [2.40,3.28] |
| ASC_1_ (%) | U[1,95] | 15.35 [8.81,23.31] | 11.06 [6.39,22.24] | 16.16 [9.37,30.68] | 14.73 [7.26,22.85] | 21.67 [13.43,33.20] | 11.48 [6.72,18.67] | 12.96 [6.51,26.21] |
| First turning point in ASC (τ_ASC1_) | U[10 May,19 Jun 2021] | 21 [18, 25] May 2021 | 21 [18,25] May 2021 | 21[18,25] May 2021 | 21[18,25] May 2021 | 21[18, 25]May 2022 | 21[18,26] May 2021 | 21[18,25] May 2021 |
| ASC_2_ (%) | U[1,95] | 1.79 [1.45,2.19] | 1.86 [1.51,2.27] | 1.65 [1.32,2.06] | 1.78 [1.43, 2.21] | 1.64 [1.31,2.04] | 1.83 [1.49,2.30] | 1.76 [1.39,2.25] |
| Second turning point in ASC (τ_ASC2_) | U[20 Jun,15 Dec 2021] | 14 [11,17] Sep 2021 | 14[12,17] Sep 2021 | 13 [7,16] Sep 2021 | 14[11,17] Sep 2021 | 10[6,14] Sep 2021 | 15[12,17] Sep 2021 | 14[11,16] Sep 2021 |
| ASC_3_ (%) | U[1,95] | 4.95 [4.67,5.17] | 5.36 [5.03,5.71] | 4.40 [4.15,4.63] | 4.91 [4.66,5.17] | 4.17 [3.97,4.35] | 5.23 [4.89,5.56] | 5.00 [4.64,5.24] |
| Third turning point in ASC (τ_ASC3_) | U[1 Jan,5 Apr 2022] | 15 [13,16] Mar 2022 | 14 [13,16] Mar 2022 | 15[13,17] Mar 2022 | 15 [13,16] Mar 2022 | 16 [14, 17] Mar 2022 | 14[13,16] Mar 2022 | 15[13,16] Mar 2022 |
| ASC_4_ (%) | U[1,95] | 19.91 [15.61,27.23] | 23.49 [17.73,30.86] | 16.93 [13.34,22.53] | 20.89 [16.73,26.77] | 18.12 [13.50,25.46] | 22.59 [17.65,29.50] | 21.41 [16.05,27.75] |
| CFR of Alpha variant (%)^♦^ | U[cfr0/5,5cfr0] | 0.38 [0.22, 0.63] | 0.37 [0.21,0.58] | 0.39 [0.23,0.63] | 0.38 [0.22,0.61] | 0.43 [0.23,0.62] | 0.37 [0.20,0.59] | 0.37 [0.20,0.61] |
| CFR of Delta variant (%)^♦^ | U[cfr0/5,5cfr0] | 1.05 [0.98,1.14] | 1.06 [0.97,1.14] | 1.05 [0.96,1.14] | 1.05 [0.97,1.14] | 1.02 [0.94,1.11] | 1.06 [0.96,1.15] | 1.05 [0.96,1.13] |
| CFR of Omicron variant (%)^♦^ | U[cfr0/5,4cfr0] | 0.29 [0.22,0.37] | 0.27 [0.20,0.34] | 0.30 [0.23,0.38] | 0.29 [0.22,0.37] | 0.33 [0.26,0.40] | 0.27 [0.19,0.341] | 0.28 [0.21,0.36] |
| Dispersion parameter for cases (η_Case_) | U[5,200] | 31.864 [27.76,37.11] | 31.60 [27.24,36.60] | 32.14 [28.00,38.64] | 32.02 [27.70,37.29] | 34.34 [29.81,39.83] | 32.39 [28.40,37.66] | 32.23 [28.22,37.58] |
| Dispersion parameter for deaths (η_Death_) | U[1.001,5.00] | 1.005 [1.002,1.022] | 1.005 [1.002,1.019] | 1.004 [1.002,1.016] | 1.006 [1.002,1.017] | 1.006 [1.002,1.022] | 1.005 [1.002,1.020] | 1.005 [1.002,1.018] |
| VE of 1 dose against Omicron e_1,ο_ (%) | U[5,50] | 44.42 [22.28,49.83] | 44.30 [19.49, 49.79] | 45.46 25.32,49.75] | 46.03 [26.80,49.80] | 44.55 [20.84,49.72] | 45.57 [25.86,49.85] | 45.65 [24.11,49.86] |
| VE of full doses against Omicron e_2,ο_ (%) | U[10,75] | 72.22 [58.45,74.89] | 72.11 [61.60,74.91] | 72.36 [61.34,74.93] | 72.59 [64.30,74.82] | 71.90 [59.08,74.86] | 73.20 [66.43,74.93] | 72.20 [59.90,74.89] |
| Replacement rate of Alpha by Delta variant (C_δ_) | U[0.05,2.0] | 0.077 [0.059,0.101] | 0.062[0.052,0.087] | 0.087 [0.062,1.63] | 0.074 [0.06,0.100] | 0.808 [0.102,1.938] | 0.068 [0.053,0.106] | 0.073 [0.052,0.103] |
| Replacement rate of Delta by Omicron variant (C_ο_) | U[0.05,2.0] | 0.147 [0.114,0.192] | 0.124 [0.091,0.165] | 0.18 [0.134,0.249] | 0.144 [0.111,0.194] | 0.304 [0.203,0.884] | 0.277 [0.094,1.787] | 0.137 [0.105,0.182] |
| DIC | – | 5385.68 | 5526.31 | 5563.03 | 5481.14 | 5731.38 | 5482.42 | 5417.42 |

^♦^cfr0 = 0.44% was the naïve estimate by dividing the total number of deaths by the total of cases reported.


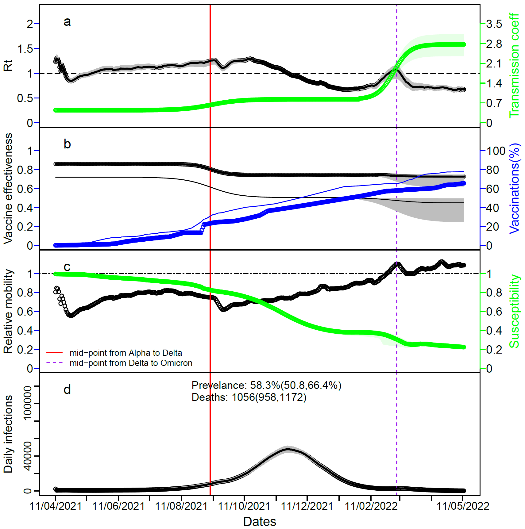

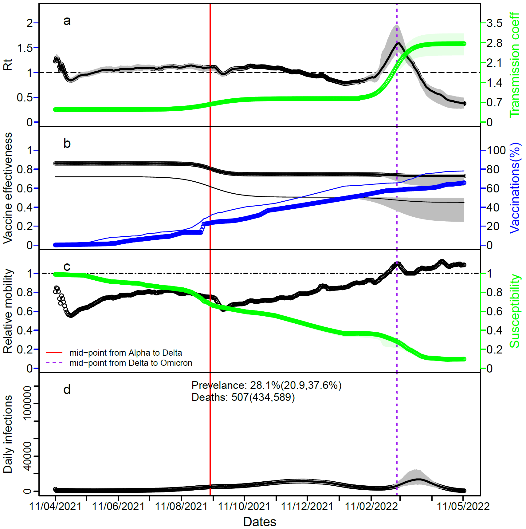

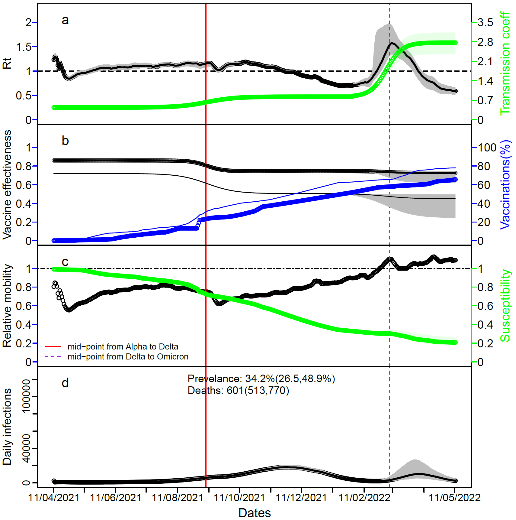

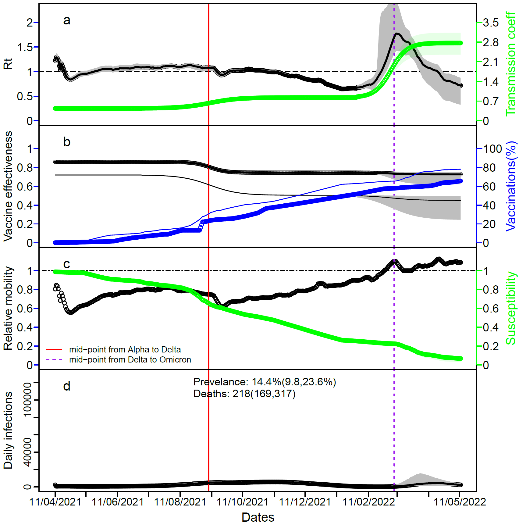

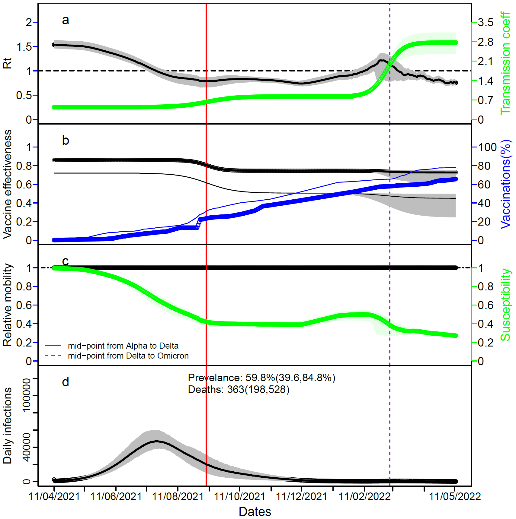

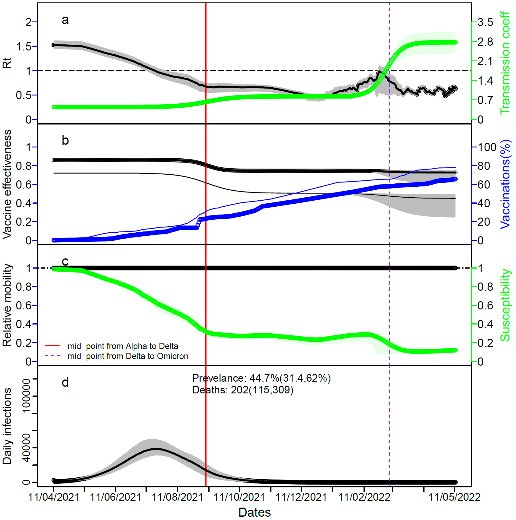

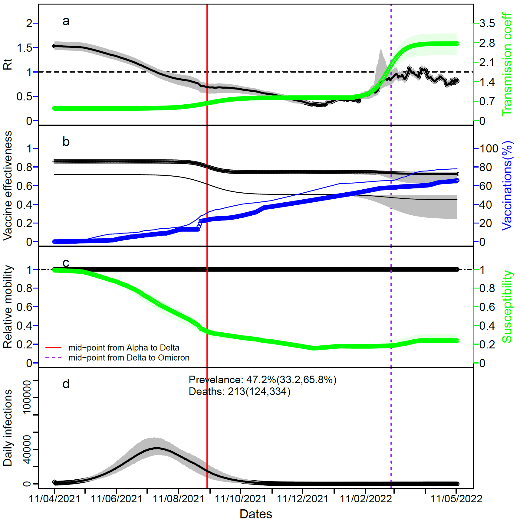

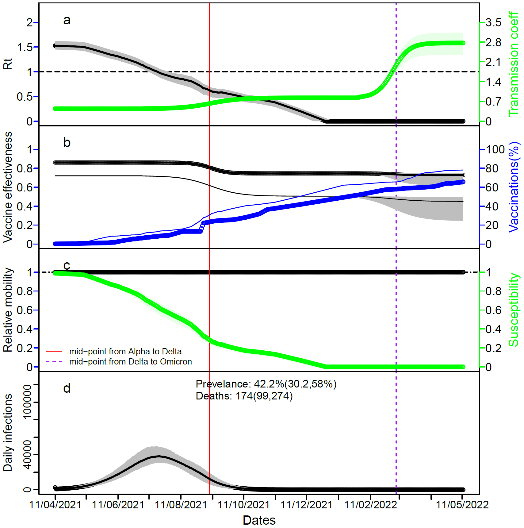


Fig S4 Impact of NPIs and vaccinations on transmissibility and susceptibility and outbreaks of SARS-CoV-2 infections in Laos. Eight situations were considered: situation with behaviour change and VE halved (top 1^st^ column); with behaviour change and VE=100% (top 2^nd^ column); with behaviour change and life-long immunity (top 3^rd^ column); with behaviour change and perfect life-long immunity (top 4^th^ column); situation without behaviour change but VE halved (bottom 1^st^ column); No behaviour change but VE=100% (bottom 2^nd^ column); No behaviour change and with life-long immunity (bottom 3^rd^ column); No behaviour change and with perfect life-long immunity (bottom 4^th^ column). The characters shown were the same as in Fig 5.

**References**

1. <https://www.google.com/covid19/mobility/>. Accessed 16 May 2022
2. Hale T, Angrist N, Goldszmidt R, Kira B, Petherick A, Phillips T, et al. A global panel database of pandemic policies (Oxford COVID-19 Government Response Tracker). Nat Hum Behav. 2021;5:529-38.
3. <https://ourworldindata.org/covid-vaccinations>. Accessed 18 May 2022.
4. [https://en.wikipedia.org/SRAS-CoV-2/](%20https://en.wikipedia.org/SRAS-CoV-2/) Accessed 20 September 2022
5. <https://covid19policy.adb.org/index.php/policy-measures/LAO> Accessed 20 March 2023
6. <https://www.who.int/laos/emergencies/covid-19-in-lao-pdr/situation-reports>. Accessed 15 March 2023
7. Rabaan AA, Mutair AA, Hajissa K, Alfaraj AH, Al-Jishi JM, Alhajri M, et al. A comprehensive review on the current vaccines and their efficacies to combat SARS-CoV-2 variants. Vaccine 2022;10(10):1655.
8. Zeng B, Gao L, Zhou Q, Yu K, Sun F. Effectiveness of COVID-19 vaccines against SARS-CoV-2 variants of concern: a systematic review and meta-analysis. BMC Med. 2022;20:200.
9. Jin L, Li Z, Zhang X, Li J, Zhu F. CoronaVac: A review of efficacy, safety, and immunogenicity of the inactivated vaccine against SARS-CoV-2. Hum vaccin immunother. 2022:18(6):e2096970.
10. Hall VJ, Foulkes S, Saei A, Andrews N, Oguti B, Charlett A, et al. COVID-19 vaccine coverage in health-care workers in England and effectiveness of BNT162b2 mRNA vaccine against infection (SIREN): a prospective, multicentre, cohort study. Lancet 2021;397:1725-35.
11. Andrews N, Stowe J, Kirsebom F, Toffa S, Rickeard T, Gallagher E, et al., COVID-19 vaccine effectiveness against the Omicron (B. 1.1. 529) variant. N Engl J Med. 2022; 386:1532-46.
12. Pouwels K, Pritchard E, Matthews PC, Stoesser N, Eyre DW, Vihta KD, et al. Effect of Delta variant on viral burden and vaccine effectiveness against new SARS-CoV-2 infections in the UK. Nat Med. 2021;27:2127-35.
13. Tsang NNY, So HC, Cowling BJ, Leung GM, Ip DKM. Effectiveness of BNT162b2 and CoronaVac COVID-19 vaccination against asymptomatic and symptomatic infection of SARS-CoV-2 omicron BA.2 in Hong Kong: a prospective cohort study. Lancet Infect Dis. 2023;23:421-34.
14. McMenamin ME, Nealon J, Lin Y, Wong JY, Cheung JK, Lau EHY, et al. Vaccine effectiveness of one, two, and three doses of BNT162b2 and CoronaVac against COVID-19 in Hong Kong: a population-based observational study. Lancet Infect Dis. 2022;22:1435–43.
15. Corchado-Garcia J, Zemmour D, Hughes T, Bandi H, Cristea-Platon T, Lenehan P, et al. Analysis of the effectiveness of the Ad26.COV2.S adenoviral vector vaccine for preventing COVID-19. JAMA Network Open 2021;4(11):e2132540.
16. Polinski JM, Weckstein AR, Batech M, Kabelac C, Kamath T, Harvey R, et al. Durability of the single dose Ad26.COV2.S vaccine in the prevention of COVID-19 infections and hospitalizations in the US before and during the Delta variant surge. JAMA New Open 2022;5(3):e222959.
17. Jara A, Undurraga EA, Gonzalez C, Paredes F, Fontecilla T, Jara G, et al. Effectiveness of an Inactivated SARS-CoV-2 Vaccine in Chile. N Engl J Med. 2021;385:875-84.
18. Vokó Z, Kiss Z, Surjan G, Surjan O, Barcza Z, Palyi B, et al. National effectiveness of five SARS-CoV-2 vaccines in Hungary – The HJUN-VE study. Clin Microbiol Infect. 2022;28(3):398-404.
19. Bello-Chavolla OY, Antonio-Villa NE, Valdes-Ferrer SI, Fermin-Martinez CA, Fernandez-Chirino L, Vargas-Vazquez A, et al. 2023. Effectiveness of a nation-wide COVID-19 vaccination program in Mexico against symptomatic COVID-19, hospitalization, and death: a retrospective analysis of national surveillance data. Int J Infect Dis. 2023;129:188-96.
20. Gram MA, Embog HD, Schelde AB, Friis NU, Nielsen KF, Moustsen-Helms IR, et al. Vaccine effectiveness against SARS-CoV-2 infection or COVID-19 hospitalization with the Alpha, Delta, or Omicron SARS-CoV-2 variant: A nationwide Danish cohort study. PLoS Med. 2022;19(9):e1003992.
21. Can G, Acar HC, Aydin SN, Balkan II, Karaali R, Budak B, et al. Waning effectiveness of CoronaVac in real life: a retrospective cohort study in health care workers. Vaccine 2022;40(18):2574–79.
22. Feikin DR, Higdon MM, Abu-Raddad LJ, Andrews N, Araos R, Goldberg Y, et al. Duration of effectiveness of vaccines against SARS-CoV-2 infection and COVID-19 disease: results of a systematic review and meta-regression. Lancet 2022;399:924-44.
23. Cohen BA, Cirillo PM, Murphy CC, Krigbaum NY, Wallace AW. SARS-CoV-2 vaccine protection and deaths among US veterans during 2021. Science 2022;375:331-6.
24. Suah JL, Hush M, Tok PSK, Tng BH, Thevananthan T, Low EV, et al. Waning COVID-19 vaccine effectiveness for BNT162b2 and CoronaVac in Malaysia: an observational study. Int J Infect Dis. 2022;119:69–76.
25. Tartof SY, Slezak JM, Fischer H, Hong V, Ackerson BK, Ranasinghe ON, et al. Effectiveness of mRNA BNT162b2 COVID-19 vaccine up to 6 months in a large integrated health system in the USA: a retrospective cohort study. Lancet 2021;398: 1407-16.
26. Lau JJ, Cheng SM, Leung K, Lee CK, Hachim A, Tsang LCH, et al. Real-world COVID-19 vaccine effectiveness against the Omicron BA.2 variant in a SARS-CoV-2 infection-naive population. Nat Med*.* 2023;29:348-57.
27. Wearing HJ, Rohani P, Keeling MJ. Appropriate models for the management of infectious diseases. PLoS Med. 2005;2(7):e174.
28. Birrell P, Ketsetzis G, Gay NJ, Cooper BS, Presani AM, Harris R, et al*.* Bayesian modelling to unmask and predict influenza A/H1N1pdm dynamics in London. Proc. Natl. Acad. Sci. USA 2011;108:18238–43.
29. [Laos Population (2020) – Worldometer (worldometers.info)](https://www.worldometers.info/world-population/laos-population/) <https://www.worldometers.info/world-population/laos-population>. Accessed 16 August 2022
30. Eyre DW, Taylor D, Purver M, Chapman D, Fowler T, Pouwels KB, et al. Effect of COVID-10 vaccination on transmission of Alpha and Delta variants. N Engl J Med. 2022;386:744-56.
31. Roberts MG, Nishiura H. Early Estimation of the Reproduction Number in the Presence of Imported Cases: Pandemic Influenza H1N1-2009 in New Zealand. PLOS One 2011;6(5):e17835.
32. Lopez Bernal J, Panagiotopoulos N, Byers C, Vilaplana TG, Boddington N, Zhang X-S, et al. Transmission dynamics of COVID-19 in household and community settings in the United Kingdom, January to March 2020. Euro surveill. 2022;27:2001551.
33. Zhang X-S, Xiong H, Chen Z, Liu W. Importation, local transmission, and model selection in estimating the transmissibility of COVID-19: the outbreak in Shaanxi as a case study. Trop Med Infect Dis. 2022;7:227.
34. [https://www.who.int/laos/emergencies/covid-19-in-lao-pdr/situation-reports. Accessed 20 May 2022](https://www.who.int/laos/emergencies/covid-19-in-lao-pdr/situation-reports.%20Accessed%2020%20May%202022) results from Institute Pasteur du Laos (IPL) and Lao-Oxford-Mahosot Hospital-Wellcome Trust Research Unit (LOMWRU)
35. Dankwa EA, Donnelly CA, Brouwer AF, Zhao R, Montgomery MO, Wang MK, et al. Estimating vaccination threshold and impact in the 2017-2019 hepatitis A virus outbreak among persons experiencing homelessness or who use drugs in Louisville, Kentucky, United states*.* Vaccine 2021;39:7182-90.
36. Li Q, Guan X, Wu P, Wang X, Zhou L, Tong Y, et al*.* Early Transmission Dynamics in Wuhan, China, of Novel Coronavirus-Infected Pneumonia. N Engl J Med. 2020; 382:1199-207.
37. Alene M, Yismaw L, Assemie MA, Ketema DB, Gietaneh W, Birhan TY. Serial interval and incubation period of COVID-19: A systematic review and meta-analysis. BMC Infect Dis. 2021;21:257.
38. Xu X, Wu Y, Kummer AG, Zhao Y, Hu Z, Wang Y, et al. Assessing changes in incubation period, serial interval, and generation time of 2 SARS-CoV-2 variants of concern: a systematic review and meta-analysis. BMC Med. 2023;21:374.
39. Read JM, Bridgen JRE, Cummings DAT, Ho A, Jewell CP. Novel coronavirus 2019-nCoV: early estimation of epidemiological parameters and epidemic predictions. Philos Trans R Soc Lond B Biol Sci. 2021;376:20200265.
40. Hall VJ, Foulkes S, Charlett A, Atti A, Monk EJM, Simmons R, et al. SARS-CoV-2 infection rates of antibody-positive compared with antibody-negative health-care workers in England: A large, multicentre, prospective cohort study (SIREN). Lancet 2021;397:1459–69.
41. Hansen CH, Michlmayr D, Gubbels SM, Mølbak K, Ethelberg S. Assessment of protection against reinfection with SARS-CoV-2 among 4 million PCR-tested

individuals in Denmark in 2020: a population-level observational study. Lancet 2021;397:1204–12.

1. Layton AT, Sadria M. Understanding the dynamics of SARS-CoV-2 variants of concern in Ontario, Canada: a modeming study. Sci Rep. 2022;12:2114.
2. [Sarracino](https://sites.google.com/site/fsarracino/) F, [Greyling](https://www.uj.ac.za/members/prof-talita-dalton-greyling/) T, [O'Connor](https://www.iza.org/person/30771) KJ, [Peroni](https://statistiques.public.lu/en/actors/statec/organisation/red/Peroni/index.html) C, [Rossouw](http://www.aut.ac.nz/study-at-aut/study-areas/business/research/research-areas/listings/business-economics/dr-stephanie-rossouw) S. March 2022. IZA DP No. 15171: Trust Predicts Compliance with COVID-19 Containment Policies: Evidence from Ten Countries Using Big Data.
3. Lopez Bernal J, Andrews N, Gower C, Robertson C, Stowe J, Tessier E, et al. Effectiveness of the Pfizer-BioNTech and Oxford-AstraZeneca vaccines on covid-19 related symptoms, hospital admissions, and mortality in older adults in England: test negative case-control study. BMJ 2021;373:n1088.
4. Thompson MG, Burgess JL, Naleway AL, Tyner HL, Yoon SK, Meece J, et al Interim estimates of vaccine effectiveness of BNT162b2 and mRNA-1273 COVID-19 vaccines in preventing SARS-CoV-2 infection among health care personnel, first responders, and other essential and frontline workers—Eight US location, December 2020-March 2021. *MMWR* 2021;70(13):495–500.
5. Amit S, Regev-Yochay G, Afek A, Kreiss Y, Leshem E. Early rate reductions of SARS-CoV-2 infection and COVID-19 in BNT162b2 vaccine recipients. Lancet 2021:397:875–7.
6. Verity R, Okell LC, Dorigatti I, Winskill P, Whittaker C, Imai N, et al*.* Estimates of the severity of coronavirus disease 2019: a model-based analysis. Lancet Infect. Dis. 2020:20(6):669–77.
7. R Core Team. R: A language and Environment for Statistical Computing. Vienna Austria; 2018 <http://www.R-project.org/>
8. Spiegelhalter DJ, Best N, Carlin BP, van der Linde A. Bayesian measures of model complexity and fit. J Roy Stat Soc B. 2002;64:583–639.
9. Brand SPC, Ojal J, Aziza R, Were V, Okiro EA, Kombe IK, et al. COVID-19 transmission dynamics underlying epidemic waves in Kenya. Science 2021;374:989-94.
10. Zhang X-S, Luo H, Charlett A, DeAngelis D, Liu W, Vickerman P, et al. R code and collated data for implementing Laos COVID-19 modelling: **https://github.com/wlx0871/laosCOVID19modelling.**
